# Tomonaga–Luttinger liquid behavior and spinon confinement in YbAlO_3_

**DOI:** 10.1038/s41467-019-08485-7

**Published:** 2019-02-11

**Authors:** L. S. Wu, S. E. Nikitin, Z. Wang, W. Zhu, C. D. Batista, A. M. Tsvelik, A. M. Samarakoon, D. A. Tennant, M. Brando, L. Vasylechko, M. Frontzek, A. T. Savici, G. Sala, G. Ehlers, A. D. Christianson, M. D. Lumsden, A. Podlesnyak

**Affiliations:** 1 Neutron Scattering Division, Oak Ridge National Laboratory, Oak Ridge, TN 37831 USA; 2 Department of Physics, Southern University of Science and Technology, 518055 Shenzhen, China; 3 Max Planck Institute for Chemical Physics of Solids, Nöthnitzer Str. 40, 01187 Dresden, Germany; 4 Institut für Festkörper- und Materialphysik, Technische Universität Dresden, 01069 Dresden, Germany; 5 Department of Physics and Astronomy, The University of Tennessee, Knoxville, TN 37996 USA; 6 Westlake Institute of Advanced Study, 310024 Hangzhou, P. R. China; 7 Theoretical Division, T-4 and CNLS, Los Alamos National Laboratory, Los Alamos, NM 87545 USA; 8 Shull-Wollan Center, Oak Ridge National Laboratory, Oak Ridge, TN 37831 USA; 9 Condensed Matter Physics and Materials Science Division, Brookhaven National Laboratory, Upton, NY 11973 USA; 10 Materials Science and Technology Division, Oak Ridge National Laboratory, Oak Ridge, TN 37831 USA; 11 Lviv Polytechnic National University, Lviv, 79013 Ukraine; 12 Neutron Technologies Division, Oak Ridge National Laboratory, Oak Ridge, TN 37831 USA

## Abstract

Low dimensional quantum magnets are interesting because of the emerging collective behavior arising from strong quantum fluctuations. The one-dimensional (1D) *S* = 1/2 Heisenberg antiferromagnet is a paradigmatic example, whose low-energy excitations, known as spinons, carry fractional spin *S* = 1/2. These fractional modes can be reconfined by the application of a staggered magnetic field. Even though considerable progress has been made in the theoretical understanding of such magnets, experimental realizations of this low-dimensional physics are relatively rare. This is particularly true for rare-earth-based magnets because of the large effective spin anisotropy induced by the combination of strong spin–orbit coupling and crystal field splitting. Here, we demonstrate that the rare-earth perovskite YbAlO_**3**_ provides a realization of a quantum spin *S* = 1/2 chain material exhibiting both quantum critical Tomonaga–Luttinger liquid behavior and spinon confinement–deconfinement transitions in different regions of magnetic field–temperature phase diagram.

## Abstract (web summary)

Low dimensional quantum magnetic excitations are intriguing but the experimental realizations are challenging. Here, the authors demonstrate Tomonaga–Luttinger behavior and spinon confinement in rare-earth perovskite YbAlO_3_ by inelastic neutron scattering measurements.

## Introduction

The spin *S* = 1/2 antiferromagnetic Heisenberg Hamiltonian is one of the simplest models of condensed matter physics containing fractional quantum number excitations. The phase diagram of this model includes both quantum critical (for easy-plane anisotropy) and gapped phases (for the easy-axis anisotropy). Spinons are topological excitations which can be pictured as domain walls between different Néel ground states of the system (this picture is especially appropriate for the moments with easy-axis anisotropy). In view of its exotic physics, it is exciting to find realizations of this model and it is particularly exciting to find them in unexpected places. The three-dimensional metallic system Yb_2_Pt_2_Pb is a recent example, where the neutron scattering revealed the existence of one-dimensional spinon continuum^1,2^. The unusual properties of Yb_2_Pt_2_Pb were initially attributed to its peculiar crystal structure^3,4^, but more recent work^1^ demonstrated that the peculiar form of exchange interactions in rare earth ions plays a dominant role. However, the metallic nature of this material complicates the analysis of its magnetic properties.

Here we discuss the insulating analog of Yb_2_Pt_2_Pb, namely Yb-based quasi-1D quantum magnet, YbAlO_3_^5^. RKKY interactions are not present because YbAlO_3_ is an insulator. Despite the strong uniaxial single-ion anisotropy of the Yb magnetic moments, our neutron scattering data demonstrate that, unlike Yb_2_Pt_2_Pb, YbAlO_3_ is described by a nearly isotropic (Heisenberg) intrachain interaction, which results in Tomonaga–Luttinger liquid behavior^6–8^ over a finite window of applied magnetic field values. Below the Néel temperature *T*_N_ = 0.88 K, the ordered moments from neighboring chains produce a staggered molecular field that confines the fractional spinon excitations. An additional advantage of YbAlO_3_ is that the moments can be saturated with a relatively low magnetic field of 1.1 T, enabling exploration of the entire magnetic field–temperature phase diagram.

## Results

### Magnetization, single-ion anisotropy, and two magnetic sublattices

YbAlO_3_ crystallizes in an orthorhombically distorted perovskite structure^9^, with room temperature lattice constants *a* = 5.126 Å, *b* = 5.331 Å, and *c* = 7.313 Å (in conventional *Pbnm* notation). The local point-group symmetry of the Yb^3+^ ions splits the eight-fold degenerate *J* = 7/2 (*L* = 3, *S* = 1/2) ground state multiplet (2*J* + 1 = 8) into four doublet states. The ground doublet state *m*_*J*_ = ±7/2 with small admixture of other states is well separated from the first excited levels^5^. This ensures that the low temperature and low field magnetic properties can be described by a pseudospin 1/2 model.

**Fig. 1:**
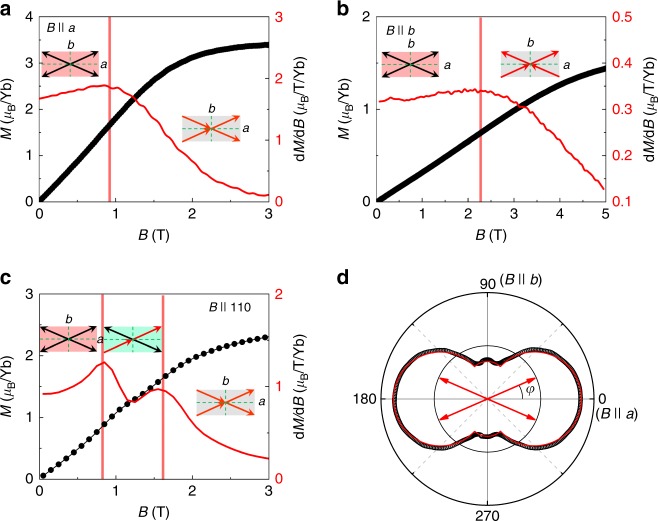
Single-ion anisotropy of YbAlO_3_. **a**–**c** Field-dependent magnetization *M* (black circles) and magnetic susceptibility d*M*/d*B* (red line) of YbAlO_3_, measured at *T* = 2 K with field along the axes *B* || *a*, *B* || *b* and *B* || [110], as indicated. The insets are sketches of the magnetic moment configurations in different field ranges. **d** Angle-dependent magnetization measured at *T* = 2 K and *B* = 5 T. Red arrows schematically show a moment configuration of Yb^3+^ at zero field with angle *φ* = 23.5° between the *a*-axis and the Yb magnetic moment. The solid line is the calculation, as described in the text

Crystalline electrical field (CEF) calculations and magnetization measurements reveal strong uniaxial (Ising-like) single-ion anisotropy, with easy-axis in the *ab*-plane. Figure 1a–c shows the field dependence of magnetization, *M*, measured at *T* = 2 K. At *B* = 5 T, the angle-dependent magnetization (Fig. 1d) is well described by the function

1 $$M \approx \frac{{M_{\mathrm{s}}}}{2}\left( {\left| {{\mathrm{cos}}(\theta - \varphi )} \right| + \left| {{\mathrm{cos}}(\varphi + \theta )} \right|} \right),$$

where *φ* = 23.5° is the tilting angle of the local easy-axis relative to the *a*-axis, *θ* indicates the direction of the applied field in the *ab*-plane, and *M*_s_ = 3.8 *μ*_B_/Yb is the saturation moment^5^. The effective *g*-factors from magnetization measurements are: *g*^*xx*^ ≃ *g*^*yy*^ = 0.46, and *g*^*zz*^ = 7.6 (ref. ^5^), which are consistent with early studies by Mössbauer effect and electron paramagnetic resonance measurements^10,11^.

In addition to the *g*-tensor anisotropy, the field-dependent magnetization reveals that the coupling between Ising moments with different tilting angles ±*φ* is weak: only one transition is observed for *B* || *a*, *B* || *b* (Fig. 1a, b), while two successive transitions are found for *B* || [110] (Fig. 1c), suggesting that magnetic moments with different easy-axis orientation can be individually tuned.

### Model Hamiltonian and zero-field inelastic neutron scattering spectrum

The most important aspect of the Yb-physics is the exchange interaction. Contrary to naive expectations, the Yb Kramers doublets do not behave as classical Ising spins when they interact with each other. The exchange processes between the pseudospins on the same chain include spin flip terms of quantum origin. The reason for this was described in ref. ^1^: the super-exchange interaction between total angular momenta of rare earth ions includes matrix elements between all eigenstates, not just between those differing by *δJ*^*z*^ = ±1. Being projected on the lowest Kramers doublet, this interaction acquires the familiar *S*^*a*^*S*^*a*^ form. We remind the reader that spin *S* = 1/2 operators *S*^*a*^ act on the Kramers doublet and, to a very good approximation, only *S*^*z*^ is linearly related to the total angular moment: *J*^*z*^ = *g*^*zz*^*S*^*z*^ with *g*^*zz*^ ≃ 7.6 $\left( {g^{xx} \simeq g^{yy} \ll g^{zz}} \right)$.

**Fig. 2:**
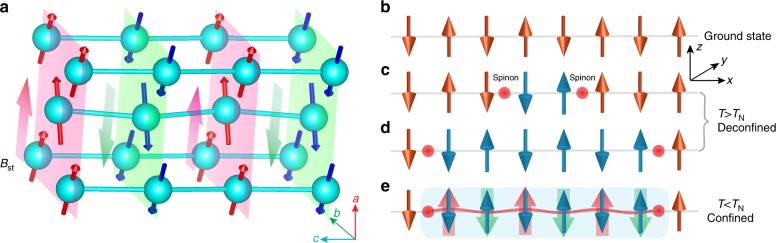
Illustration of the magnetic structure and spinon confinement in YbAlO_3_. **a** Magnetic structure below *T*_N_ = 0.88 K, where the Yb Ising moments align antiferromagnetically (AFM) in chains along the *c*-axis. These one-dimensional (1D) chains are ferromagnetically (FM) coupled in *ab*-plane, which results in a net staggered field (*B*_st_), as indicated by the large red and blue arrows. **b**–**e** Sketch of the spinon generation and confinement in a 1D AFM chain. Since only longitudinal fluctuations of Δ*S*^*z*^ = 0 are observed in YbAlO_3_, even numbers of Yb moments are flipped in the AF ground state (**b**), creating pairs of spinons (**c**). These spinons can propagate freely (deconfined) along the chain at *T* > *T*_N_ (**c**, **d**), while in the presence of the effective staggered field at *T* < *T*_N_, propagation of the spinons costs energy (confined) as the separation increases (**e**)

The magnetic coupling between different sublattices is dominated by the long-range dipole–dipole interaction, which results in a weak ferromagnetic (FM) Ising-like coupling of type $S_i^zS_j^z$ in terms of the effective spins (see Supplementary Note 2). In contrast, the intrachain interaction between neighboring spins is dominated by antiferromagnetic (AFM) super-exchange. This combination leads to an *AxGy*-type AFM ordering^5^ below the Néel temperature *T*_N_ = 0.88 K (see Fig. 2a). Above *T*_N_, the weaker dipole–dipole interchain interaction can be neglected, since thermal fluctuations have destroyed the interchain ordering. In this free-standing 1D chain picture, the elementary excitations are fractional spinons, instead of the conventional magnons (*S* = 1) of the magnetically ordered state. As illustrated in Fig. 2b–d, a pair of domain walls can propagate along the chain direction, behaving as free spin 1/2 spinons for *T* > *T*_N_. Below *T*_N_, the staggered interchain molecular field (*B*_st_) produces a confining potential that increases linearly in the distance between both spinons (Fig. 2e). Consequently, spinons get confined into bound states at temperatures below *T*_N_^12^.

**Fig. 3:**
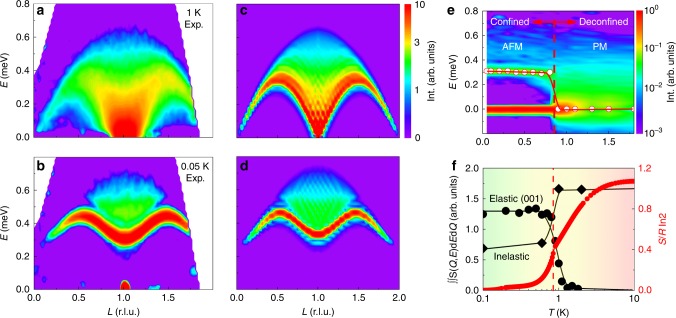
Spinon confinement of YbAlO_3_ in zero field. Inelastic neutron scattering spectra with background corrected measured at 1 K (**a**) and 0.05 K (**b**), compared to DMRG calculation with *B*_st_/*J* = 0 (**c**), and *B*_st_/*J* = 0.27 (**d**). The DMRG calculation is performed on a 64-site periodic chain with bond dimension *M* = 400 states at *T* = 0, with intrachain interaction *J* = 0.21 meV. In **d**, the elastic peak at *L* = 1 has been removed by hand. **e** Temperature evolution of the energy-dependent neutron scattering spectrum integrated in window *H* = [−0.1, 0.1] r.l.u., *K* = [−0.1, 0.1] r.l.u., *L* = [0.9, 1.1] r.l.u. A gapless continuum is observed at high temperatures, whereas a gap of about 0.3 meV (red circles) opens just at *T*_N_, due to the appearance of the staggered field. **f** Temperature dependence of the elastic (001) magnetic peak intensity (black circles) and inelastic neutron spectrum (black diamonds) integrated in the window *H* = [−0.1, 0.1] r.l.u., *K* = [−0.1, 0.1] r.l.u., *L* = [0, 2.0] r.l.u., and *E* = [0.1, 0.6] meV. The elastic and inelastic scatterings correspond to the statically ordered and fluctuating magnetic moments, respectively. The red circles are the temperature-dependent entropy (*S*). The solid lines are a guide to the eyes, and the vertical red dashed line indicates the spinon confinement–deconfinement transition at *T*_N_

In view of these considerations, the magnetic properties of YbAlO_3_ can be described with an effective 1D spin-1/2 Hamiltonian

2 $${\cal H} = J\mathop {\sum}\limits_i {\kern 1pt} {\boldsymbol{S}}_i \cdot {\boldsymbol{S}}_{i \ + \ 1} - B_{{\mathrm{st}}}\mathop {\sum}\limits_i ( - 1)^iS_i^z - B_{{\mathrm{ex}}}\mathop {\sum}\limits_i {\kern 1pt} S_i^z,$$

where *x*, *y*, and *z* are defined in Fig. 2b, *J* is the intrachain Heisenberg exchange, *B*_st_ is the effective staggered molecular field generated below *T*_N_, and *B*_ex_ is the external magnetic field applied along the local moments easy *z*-axis. The pseudospin *S* = 1/2 operators *S*^*a*^ act on the Kramers doublet, consisting of the *m*_*J*_ = ±7/2 states to a good approximation, which explains the *g*-tensor anisotropy. In other words, the magnetic field and the magnetic moment of the neutron can couple only to the diagonal operator *S*^*z*^ and the INS spectrum of YbAlO_3_ is dominated by longitudinal fluctuations: the very weak *g*-factor along the transverse directions, (*g*^*xx*^/*g*^*zz*^)^2^ ≈ 1/273, renders transverse fluctuations, *S*^*xy*^(***Q***, *E*), “hidden” to neutrons. Thus, the magnetic excitations observed in YbAlO_3_ only arise from longitudinal fluctuations, as demonstrated by the neutron polarization factor presented in Supplementary Note 4, and by all the simulated spectra shown in Fig. 3c, d. As we will see later, the exchange anisotropy turns out to be surprisingly small in this material. This unexpected situation encountered in an increasing number of compounds^1,13,14^ has been analyzed theoretically in ref. ^1^ and later in greater detail in ref. ^13^.

Single crystal inelastic neutron scattering measurements have been performed to match the Hamiltonian parameters to experimental observables. The zero-field spectra (*B*_ex_ = 0) are presented in Fig. 3a, b at different temperatures (see also Supplementary Note 3). Gapless spinon excitations along the chain direction (00*L*) are observed at 1.0 K (*T* > *T*_N_), with a broad two-spinon continuum extending from 0 up to about 0.7 meV in energy transfer (Fig. 3a). No gap in the spin excitation can be resolved in the paramagnetic (PM) state of YbAlO_3_ at the magnetic Brillouin zone center of ***Q*** = (001), within the instrumental resolution about 0.05 meV. Note that the temperature (1.0 K) is comparable to the instrumental resolution (0.05 meV), both of which are much smaller than the bandwidth of the two-spinon continuum in Fig. 3a, implying that we can safely exclude a scenario where the continuum arises from thermal or instrumental broadening (see also Supplementary Note 5).

In contrast, and in agreement with the analytical result for the spin-1/2 Heisenberg model in a staggered magnetic field^15^, a significant gap is observed below the ordering temperature, with a massive triplet mode and multi-spinon continuum extending from about 0.3 to 0.7 meV (Fig. 3b).

As demonstrated in ref. ^15^, the staggered field leads to strong confinement of the spinons. In the continuum limit, one can use bosonization and the Hamiltonian is reduced to the sine-Gordon model. The corresponding Lagrangian density is

3 $$\begin{array}{l}{\cal L} = \frac{1}{2}\left[ {c^{ - 1}(\partial _\tau \phi )^2 + c(\partial _x\phi )^2} \right] - \alpha B_{{\mathrm{st}}}\,{\mathrm{sin}}(\beta \phi ),\\ \beta ^2 = 2\pi ,\;c = \pi J{\mathrm{/}}2.\end{array}$$

and the spin is related to field *ϕ*(*x*):

4 $$S_n^z = \frac{\beta }{{2\pi }}\partial _x\phi + ( - 1)^nA\,{\mathrm{sin}}(\beta \phi ),$$

where *A* is a nonuniversal amplitude. Here the staggered field is determined self-consistently as the one generated by the ordered moments of neighboring chains: $B_{{\mathrm{st}}} = J_ \bot \left\langle {S_{{\mathrm{st}}}^z} \right\rangle$. The exact solution of the sine-Gordon model for this value of *β* yields the spectra $E_n(p) = \sqrt {(cp)^2 + {{\Delta }}_n^2}$ with two excitation branches corresponding to a triplet *n* = 1 and singlet *n* = 2 with ${ {\Delta }}_2 = \sqrt 3 {{\Delta }}_1,{ {\Delta }}_n\sim \left( {JB_{{\mathrm{st}}}^{\mathrm{2}}} \right)^{1/3}$. The inverse gap *c*/*Δ* ~ (*J*/*B*_st_)^2/3^ is the confinement radius. Since *B*_st_ is proportional to the order parameter, it vanishes at the transition point and the confinement radius becomes infinite. The triplet excitations with *S* = 1 are seen in the dynamical spin susceptibility.

The simulated longitudinal spin excitation spectrum is presented in Fig. 3c, d, using 64-site density matrix renormalization group calculations (DMRG) at *T* = 0 with periodic boundary conditions^16,17^. The Hamiltonian (2) captures the main features of the experimental data measured at 1 and 0.05 K for intrachain exchange *J* = 0.21 meV, staggered field *B*_st_ = 0 (Fig. 3c), and *B*_st_/*J* = 0.27 (Fig. 3d). It is worth noting that a finite temperature DMRG calculation for the same model^18^ can account for the deviations between the experimental data and our DMRG result at *T* = 0.

The nearly isotropic nature of the intrachain interaction may look surprising if we consider that the Yb^3+^ ground state doublet is highly anisotropic. However, as was pointed out in ref. ^1^, the exchange interaction of rare earth ions has a form of permutation operator which has matrix elements between all states. Being projected on the lowest Kramers doublet by the crystal field, it acquires the familiar **S**_*i*_ · **S**_*i*+1_ form. Apart from Yb_2_Pt_2_Pb and YbAlO_3_ the only known previous example of Heisenberg-like Yb chains is Yb_4_As_3_^19,20^. It should be noted, however, that the shape of the neutron scattering spectra is not sufficient to determine the magnitude of a possible easy-plane exchange anisotropy *Δ* that would still be compatible with the observed TLL at zero magnetic field: for an XXZ chain, the transverse (longitudinal) modes for 0.25 ≲ *Δ* ≲ 1 (0.5 ≲ *Δ* ≲ 1) almost do not change^21,22^. Nevertheless, as we explain in a later section, the observation of a free fermion fixed point at the saturation field *B* = *B*_s_ excludes the possibility of a significant easy-plane exchange anisotropy.

The temperature evolution of the energy-dependent scattering at the AFM zone center ***Q*** = (001) is presented in Fig. 3e. The continuous temperature-dependent entropy *S* in Fig. 3f suggests that a second-order transition occurs at *T*_N_. The gap in spin excitations closes within a narrow temperature *T*_N_ ± 0.1 K, which is unlikely due to thermal broadening. Figure 3f shows the temperature dependence of the (001) Bragg peak intensity, indicating that static moments build up at the phase transition. Meanwhile, the integrated inelastic neutron intensity shows that about half of the total spectral weight remains inelastic below *T*_N_ (see Fig. 3f). The temperature dependence of entropy (*S*) is over plotted in Fig. 3f as well. As expected from the ground state doublets, the full entropy (*R*·ln2) is reached above *T* = 10 K; however, only about 0.4 *R*·ln2 is released at *T*_N_. This value is consistent with the neutron scattering results: about half of the magnetic moment keeps fluctuating in the ordered state.

### Magnetic field-induced quantum phase transitions

**Fig. 4:**
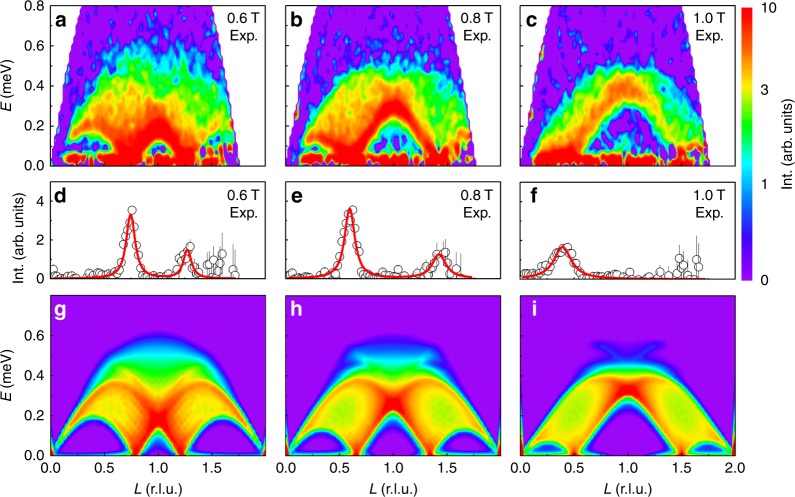
Magnetic field tuned spin excitation of YbAlO_3_. **a**–**c** Inelastic neutron scattering spectra with background corrected measured at 0.05 K, in different magnetic fields applied along the *a*-axis, of 0.6 T (**a**), 0.8 T (**b**), and 1.0 T (**c**). **d**–**f** The *Q*-cuts of in-field experimental data along wave vector (00*L*), integrated over *H* = [−0.1; 0.1] r.l.u., *K* = [−0.6; 0.2] r.l.u., and *E* = [0.05; 0.06] meV. The peak positions indicate the zero energy soft modes, where the gap closes. In the raw data, the error bar is equal to the square root of the number of counts (Poisson statistics). In the processed data, where we have various efficiency corrections and we need to account for different measurement statistics at each point, we used the rules for propagation of errors of uncorrelated measurements. **g**–**i** DMRG simulated spin excitation spectrum, with staggered field *B*_st_ = 0, and external field *B*_ex_/*J* = 0.8 (**g**), *B*_ex_/*J* = 1.2 (**h**), and *B*_ex_/*J* = 1.6 (**i**)

**Fig. 5:**
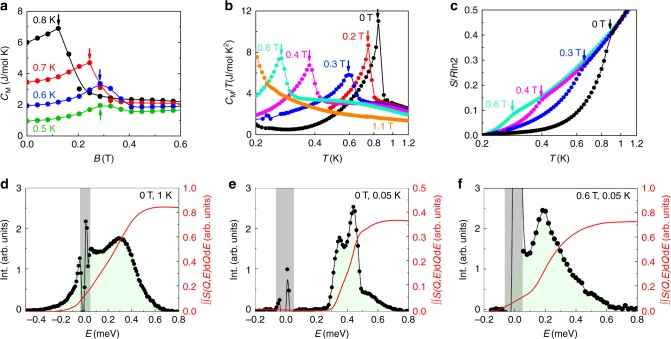
Fluctuating moments in fields. **a** Field-dependent magnetic specific heat *C*_M_/*T* at different temperatures, as indicated. **b** Temperature-dependent magnetic specific heat *C*_M_/*T* in different fields. **c** Temperature-dependent entropy, normalized to *R*·ln2, assuming doublet ground states. The arrows indicate the magnetic phase transition. **d**–**f** Energy-dependent neutron scattering intensity, integrated over wave vector *H* = [−0.2, 0.2] r.l.u., *K* = [−1, 1] r.l.u., and *L* = [0, 2] r.l.u., at different temperatures and fields, as indicated. The red lines are the integrated intensity over the energy spectrum (green shadow area) up to 0.8 meV. The gray bars indicate the instrumental resolution of about ±0.05 meV

**Fig. 6:**
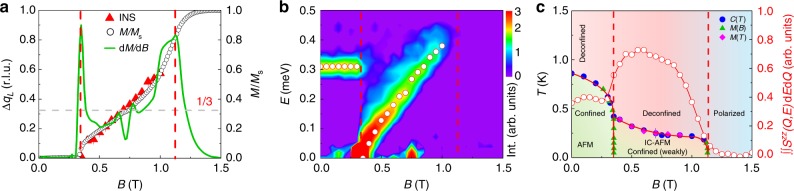
Field-dependent magnetization and magnetic phase diagram of YbAlO_3_. **a** Field-dependent zero energy soft point at wave vector shift Δ*q*_L_ = *Q*_L_ − 1, extracted from the INS. Normalized magnetization *M*/*M*_s_, measured at 0.05 K, and the corresponding magnetic susceptibility d*M*/d*B* are over plotted. *M*_s_ is the saturation moment for fields along the *a*-axis, and the horizontal dashed lines indicate the 1/3 magnetization plateau. **b** Field evolution of the energy-dependent inelastic neutron spectrum, at 0.05 K, integrated over wave vector *H* = [−0.1, 0.1] r.l.u., *K* = [−0.6, 0.2] r.l.u., *L* = [0.9, 1.1] r.l.u.. The empty circles indicate the peak positions. **c** The field-temperature (*B*−*T*) magnetic phase diagram. The field-dependent INS spectrum measured at 0.05 K is over plotted, which is integrated over *H* = [−0.1, 0.1] r.l.u., *K* = [−0.6, 0.2] r.l.u., *L* = [0, 2] r.l.u., and *E* = [0.05, 0.6] meV. The vertical dashed lines indicate the critical field *B*_c_ = 0.35 T, and saturation field *B*_s_ = 1.13 T, defined through the peak positions of d*M*/d*B*

With a magnetic field applied along the *a*-axis, the static AFM order is suppressed at the critical field *B*_c_ = 0.35 T in favor of incommensurate magnetic ordering. As entering the IC-AFM phase at *B*_c_, the spin excitation gap in the magnetic zone center ***Q*** = (001) closes abruptly (Figs. 4a–c, 5e, f and 6b). Figure 5a shows the field dependence of the magnetic contribution to the specific heat *C*_M_/*T* around *B*_c_. The step-like anomaly becomes smaller and broader upon decreasing *T* and finally evolves into a weak maximum around *B*_c_ (see also Supplementary Note 1). Meanwhile, the field-temperature (*B*–*T*) phase line becomes very steep (d*B*/d*T* → −∞) as *T* approaches 0 K (Fig. 6c). This indicates that the system is tuned through a first-order phase transition at *T* = 0 K^23^, which is consistent with the neutron scattering observations (Fig. 6b).

For *B* > *B*_c_, the longitudinal (*S*^*zz*^(***Q***, *E*)) and transverse (*S*^*xx*^(***Q***, *E*), *S*^*yy*^(***Q***, *E*)) components of the dynamical spin structure factor behave differently^24^. The bosonization formula (4) is modified in the presence of finite magnetization 〈*S*^*z*^〉 = *M*:

5 $$S_n^z = M + \frac{\beta }{{2\pi }}\partial _x\phi + A\,{\mathrm{sin}}(\beta \phi + 2\pi Mn),$$

Therefore, in the field-induced incommensurate phase, the interchain interaction becomes

6 $$\mathop {\sum}\limits_{i,j} {\kern 1pt} J_{ij}S_i^zS_j^z \to \mathop {\sum}\limits_{i,j} {\kern 1pt} J_{ij}A^2\,{\mathrm{cos}}\left[ {\beta \left( {\phi _i - \phi _j} \right)} \right].$$

The oscillatory term does not survive after summing over the phases. As we have mentioned above, the spectral gap is suppressed when the magnetic field exceeds the critical value, and the single-chain system becomes critical. However, the interchain coupling remains relevant and leading to long-range magnetic ordering and the corresponding thermodynamic phase transition (see Figs. 5b and 6c). Below the transition one can expand the cosine in (6) and obtain the quadratic action for *ϕ* so that the interaction leads to a gapless phason mode, which explains a finite low-energy spectral weight on Fig. 5f, and a significant *C*_M_/*T* values below the transition:

7 $$\omega ^2 = v^2q_{||}^2 + \mathop {\sum}\limits_j {\kern 1pt} a_j{\mathrm{sin}}^{\mathrm{2}}({\bf{qe}}_j{\mathrm{/}}2).$$

At higher energies one can again approximate $\mathop {\sum}\nolimits_j {\kern 1pt} {\mathrm{cos}}\left[ {\beta \left( {\phi _i - \phi _j} \right)} \right] \approx {\mathrm{cos}}\,\beta \phi _i\mathop {\sum}\nolimits_j \left\langle {{\mathrm{cos}}\,\beta \phi _j} \right\rangle$ to obtain the effective sine-Gordon model, as it was done in ref. ^15^. The gapped *S* = 1 excitations of the sine-Gordon model are allowed to decay into low-energy phasons and become resonances.

Figures 4a–c and 6b include the experimental spin excitation spectra of YbAlO_3_ for different external fields. The low energy features discussed above are obscured in the color pictures since the main spectral weight remains in the single chains. The ***Q***-cuts show that the incommensurate (IC) zero energy soft modes are symmetrically located around the AF zone center (Fig. 4d–f)^24^. The shift of these IC wave vectors *Q*_*L*_ = 1 ± Δ*q*_*L*_ is directly proportional to the field-induced magnetization *M*, i.e., Δ*q*_*L*_ ~ 2*πM*/*M*_s_. As one can see in Fig. 6a, the INS zero energy soft point traced the magnetization curve up to about 1.0 T. Finally, all the magnetic moments become fully polarized above the saturation field *B*_s_ = 1.1 T. As we have emphasized before, no magnon-like spectra can be observed for *B* > *B*_s_, because the modes become purely transverse.

For *B*_c_ < *B* < *B*_s_, the Yb^3+^ moments are highly fluctuating even in the ordered phase. Figure 5d–f shows the energy-dependent neutron scattering intensity integrated over the first Brillouin zone. The integrated intensity up to 0.8 meV, $S_{{\mathrm{int}}} = {\int\!\!\!\!\!\int} {\kern 1pt} S\left( {{\boldsymbol{Q}},E} \right)\;{\mathrm{d}}{\boldsymbol{Q}}{\mathrm{d}}E$, is then an estimate of the square of the fluctuating moment. In zero field, *S*_int_ at 0.05 K (AFM ordered state) is about 0.37/0.85 ≃ 44% (normalized by the intensity at *T* = 1 K and *B* = 0); while in 0.6 T, it reaches about 0.72/0.85 ≃ 85%. Similar conclusions are found in the temperature-dependent specific heat and entropy as well. The integrated temperature-dependent entropy shown in Fig. 5c reveals that only 10–20% of *R*·ln2 is released at the IC-AFM phase transition. These together suggest that large moment fluctuations coexist with a small ordered moment (10–20% of the full moment) in the IC-AFM phase (Fig. 6c).

Since the interchain molecular field is proportional to the magnitude of the ordered moments, in a first approximation we can ignore it for *B*_c_ < *B* < *B*_s_. This simplification reduces the Hamiltonian (1) to the conventional Heisenberg model in an external field *B*_ex_. We again use the DMRG calculation to obtain the longitudinal spin structure factor *S*^*zz*^(***Q***, *E*) above *B*_c_, in various external fields *B*_ex_/*J* = 0.8, 1.2, 1.6, as presented in Fig. 4g–i. We note that, although a self-consistent treatment of the static moments from neighboring chains is required to obtain a more accurate excitation spectrum, overall qualitative agreement with the experimental data is already achieved at this level of approximation.

It is interesting to note that an *M*/*M*_s_ = 1/3 magnetization plateau appears at 0.75 T, indicating the stabilization of a new magnetic structure. A rather abrupt increase of the magnetic susceptibility is also observed at *M*/*M*_s_ = 1/2, although no obvious magnetization plateau can be resolved.

A key and surprising observation of this work is that YbAlO_3_ can be modeled by nearly isotropic Heisenberg spin 1/2 chains, despite the very strong *g*-tensor anisotropy. This unusual combination together with the dominant dipolar interchain interactions make YbAlO_3_ an ideal material for studying the competition between sliding TLL’s and spin density wave ordering. Our measured phase diagram reflects such competition. Each chain behaves as a TLL at temperatures $T_{\mathrm{N}} < T \ll J$, while spin density wave ordering (SDW) develops below *T*_N_. The intrachain Hamiltonian has a divergent longitudinal magnetic susceptibility *χ*(*q*, *ω*) at *q* = 2*k*_f_ and *ω* = 0, where *k*_f_ is the Fermi wave vector of the fermionic system associated with the TLL. Given that *k*_f_ = ± (*m*/2 + 1/2)*π*, with *m* = *M*/*M*_s_, *k*_f_ increases linearly with *M* between *B*_c_ and *B*_s_ (*k*_f_ = *π*/2 for 0 ≤ *B* < *B*_c_). The Ising-like interchain dipolar interaction favors a collinear structure with maximum amplitude of the local moment at each site. However, the magnitude of the moment $\left| {\left\langle {S_i^z} \right\rangle } \right|$ is necessarily modulated for general values of 2*k*_f_, leading to competition between intrachain exchange and the interchain Ising-like dipolar interaction that penalizes any longitudinal modulation of the magnetic moments. The only exceptions are 2*k*_f_ equal to 0 (↑↑↑), *π* (↑↓↑↓) or ±2*π*/3(↑↑↓). The magnitude of the local moments is not modulated for these particular ordering wave vectors, implying that intrachain and interchain interactions can be satisfied simultaneously. Note that the ↑↑↓ ordering only contains Fourier components *q* = ±2*π*/3 plus the field-induced *q* = 0 component. The lack of competition for these special cases explains the observed magnetization plateaus at *m* = 0, 1, and 1/3. A spin density wave with dominant 2*k*_f_ ordering wave vector is expected for other magnetization values, as it is confirmed by the fact that the ordering wave vector extracted from the INS data tracks the magnetization curve (see Fig. 4a). However, we expect that higher harmonics will be induced for this magnetization values producing spin superstructures similar to those emerging from the anisotropic next-nearest-neighbor Ising (ANNNI) model^25–27^.

### Quantum critical scaling and universality class

The quantum phase transition at the saturation field is of second order, implying a quantum critical point (QCP) at *B* = *B*_*s*_ and *T* = 0. This QCP is a Gaussian fixed point because the effective dimension of the low-energy effective theory is *D* = 3 + 1 (the dynamical exponent is *z* = 1 because of the exchange anisotropy). For temperatures higher than the exchange anisotropy and the interchain coupling, we expect a crossover into a regime controlled by a free fermion fixed point (*ν* = 1/2) in dimension *D* = 1 + 2 (the dynamical exponent becomes *z* = 2 because the field couples to a conserved quantity in the absence of exchange anisotropy)^28,29^.

**Fig. 7:**
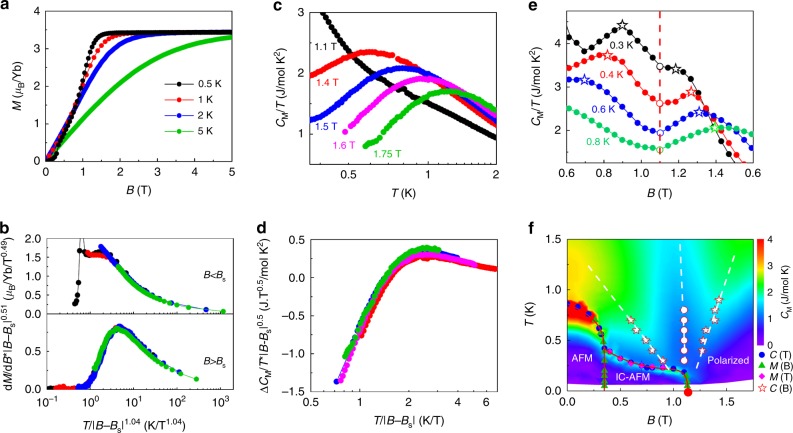
Quantum critical scaling and universality of YbAlO_3_. **a** Field-dependent magnetization *M* measured at different temperatures. **b** Critical scaling of the magnetic susceptibility in **a**, for fields both below and above *B*_s_. **c** Temperature-dependent magnetic specific heat *C*_M_/*T* measured at different fields. **d** Critical scaling of the field-dependent magnetic specific heat Δ*C*_M_/*T* shown in **c**. **e** Field-dependent magnetic specific heat *C*_M_/*T* at different temperatures. The red dashed line indicates the saturation field *B*_s_ = 1.1 T at the minimum, and crossovers with two broad maximum shoulders are observed. **f** Contour plot of the magnetic specific heat *C*_M_ as a function of temperature and field. Magnetic phase boundaries extracted through different measurements are over-plotted. The red circles indicate the saturation field *B*_s_ at different temperatures, and the empty stars indicate the crossover defined through the field-dependent specific heat maxima

Figure 7a shows the field-dependent magnetization *M* at different temperatures for *T* > 0.5 K. Near the QCP, the magnetic susceptibility scales as

8 $$\frac{{{\mathrm{d}}M}}{{{\mathrm{d}}B}} = \tilde B^{\nu (d \ + \ z) - 2}\varphi \left( {\frac{T}{{\tilde B^{\nu z}}}} \right),$$

where *d* is the spatial dimension and $\tilde B = \left| {B - B_{\mathrm{s}}} \right|$. The values *d*, *ν*, and *z* can be obtained by minimizing the deviations from this scaling behavior (see Supplementary Note 6), giving:

9 $$\nu z = 1.04,$$

10 $$2 - \nu (d + z) = 0.51.$$

As shown in Fig. 7b, this set of exponents collapses the measured susceptibility data.

In addition, the field-dependent specific heat should obey the following scaling relation^30–33^:

11 $$\frac{{{\mathrm{\Delta }}C_{\mathrm{M}}}}{T} = \tilde B^{\nu (d - z)}\psi \left( {\frac{T}{{\tilde B^{\nu z}}}} \right) = \tilde B^{ - 0.5}\psi \left( {\frac{T}{{\tilde B}}} \right),$$

where

12 $$\frac{{{\mathrm{\Delta }}C_{\mathrm{M}}}}{T} = \frac{{C_{\mathrm{M}}(B)}}{T} - \frac{{C_{\mathrm{M}}(B_{\mathrm{s}})}}{T}.$$

The measured temperature-dependent *C*_M_/*T* curves, shown in Fig. 7c, collapse into a single curve for *T* ≥ 0.3 K, once they are rescaled with *νz* = 1 and *d* = 1 (see Fig. 7d).

*C*_*M*_(*B*)/*T* exhibits a clear two-peak structure in the vicinity of the quantum critical region for each fixed temperature *T* ≥ 0.3 K (star markers in Fig. 7e). This is consistent with the expectation of the 1D TLL behavior as discussed in ref. ^34^. Defining this peak position as the crossover temperature T^*^ into the quantum critical regime, the linear dependence $T^ \ast \sim \tilde B^{\nu z}$ with *νz* = 1 for *T* ≥ 0.3 K again agrees with the free fermion fix point (Fig. 7e, f). We note that the free fermion fixed point is only compatible with an isotropic exchange interaction because the applied magnetic field is nearly orthogonal to the uniaxial symmetry chain axis (as shown in Fig. 2, the magnetic moments are nearly perpendicular to the chains). Any type of exchange anisotropy would then change the universality class of this QCP to Ising in *D* = 1 + 1 (*z* = *ν* = 1). The single-chain Hamiltonian must then be isotropic (Heisenberg) for the model to retain a U(1) symmetry in the presence of the external magnetic field. Based on the above-described analysis and the value of the exchange parameter (*J* ≈ 0.21 meV), we can narrow down the range of the exchange anisotropy to 0.88 ≲ *Δ* ≲ 1.

## Discussion

Our results demonstrate that *f*-electron-based magnets can provide realizations of various aspects of quasi-one-dimensional physics. This is so despite the presence of strong spin–orbit coupling combined with crystal field splitting that produce a ground state Kramers doublet with well separated values of *m*_J_. Naively one would think about such doublets should behave as classical (Ising) spins. This unexpected situation, which is appearing in an increasing number of Yb-based compounds^1,13,14^, enables the study of model Hamiltonians, which have traditionally been regarded as “toy models” due to the combination of interactions that do not appear together. The array of TLL’s coupled by density–density interactions is a clear example of a model that was originally introduced to study the possible existence of sliding TLL phases, but it is difficult to find in real materials due to the very unusual combination of intrachain XXZ exchange and pure Ising interchain interaction. YbAlO_3_ provides a natural realization of the spin 1/2 version of this model, enabling the observation of a quantum fractional spinon continuum above *T*_N_ and the transition into a confined (magnetically ordered) low-temperature state characterized by massive excitations. Both, the gapless spinon spectrum and the critical behavior around the saturation field at *T* > *T*_*N*_ unambiguously demonstrate that the intrachain exchange is Heisenberg-like to a very good approximation.

Rau and Gingras^13^ derived an isotropic super-exchange interaction for Yb-based compounds with edge-sharing octahedra. While it is not yet clear that this result applies directly to our material, it shows that Yb-based compounds can support nearly isotropic super-exchange of the type that we are finding in YbAlO_3_. Also, note that further experiments such as an electron spin resonance would help quantify the small anisotropies in this system.

Our observations in YbAlO_3_ suggest that it is worth exploring other members of the rare earth perovskite family RMO_3_. Compared to most of the *d*-electron-based spin chains with critical fields of the order 10–100 T, YbAlO_3_ can be saturated with a field of order 1 T owing to the much weaker exchange interaction *J* and large value of the effective *g*-factor. In addition, the crystal structure naturally adapts to existing standard perovskite thin film substrates. These properties can be exploited in future spinon-based research^35^ for material engineering under easily-accessible laboratory conditions.

## Methods

### Sample preparation and thermal property measurements

YbAlO_3_ single crystals were grown by the Czochralski technique^9^. Magnetization measurements were carried out using three magnetometers for different temperature ranges: commercial vibration sample magnetometer Quantum Design Magnetic Property Measurement System (MPMS-VSM) for the high-temperature measurements *T* = 1.8–400 K, MPMS-3 with ^3^He insert for the temperatures *T* = 0.5–4 K, and high-resolution capacitive Faraday magnetometer^36^ for the low-temperature range *T* = 0.05–0.9 K. The specific heat measurements were carried out using the relaxation time method, with a Quantum Design Physical Property Measurements System down to temperatures of 0.36 K and a custom compensated heat-pulse calorimeter with dilution refrigerator insert at MPI CPfS^37^.

### Neutron scattering

Neutron scattering measurements were performed at two fixed incident energy of 3.32 meV (*λ*_*i*_ = 4.97 Å) and 1.55 meV (*λ*_*i*_ = 7.26 Å), with the time-of-flight Cold Neutron Chopper Spectrometer^38,39^, at the Spallation Neutron Source (SNS) at Oak Ridge National Laboratory. A single crystal of YbAlO_3_ of about 0.59 g was aligned in the (0*KL*) scattering plane, with magnetic field along the vertical [100] direction. A dilution refrigerator insert that can access temperatures as low as 0.05 K was used. The software packages DAVE^40^ and MantidPlot^41^ were used for data reduction and analysis.

We applied two different methods to remove the background near |***Q***| = 0 from the direct beam. For zero field data we used an empty can file. For measurements under different fields, we used the high field data at 2 T for background subtraction (almost all the magnetic inelastic signal of the Yb Ising moments is suppressed above the saturation field of 1.1 T). As presented in Figs. 3–5, the background correction produces some uncertainty within the instrumental resolution of ±0.05 meV, but it removes the instrumental background for all the other inelastic energy range.

## Supplementary information

Supplementary Information

Peer Review File

## Data Availability

The datasets generated during the current study are available from the corresponding author on reasonable request.
