# Supplementary material for: Tomonaga–Luttinger liquid behavior and spinon confinement in YbAlO3
Source: Nat Commun. 2019 Feb 11;10:698. doi: 10.1038/s41467-019-08485-7 (PMC6370837; doi:10.1038/s41467-019-08485-7)
Supplement: Supplementary file 1 — Supplementary Information [file 41467_2019_8485_MOESM1_ESM.pdf]

Supplementary Information
Tomonaga-Luttinger Liquid Behavior and Spinon Confinement in $YbAlO_3$

L. S. Wu,[1,2] S. E. Nikitin,[3,4] Z. Wang,[5] W. Zhu,[6,7] C. D. Batista,[5,8]
A. M. Tsvelik,[9] A. M. Samarakoon,[1] D. A. Tennant,[10,8] M. Brando,[3]
L. Vasylechko,[11] M. Frontzek,[1] A. T. Savici,[1] G. Sala,[1] G. Ehlers,[12]
A. D. Christianson,[10,1] M. D. Lumsden,[1] and A. Podlesnyak[1]

[1] *Neutron Scattering Division, Oak Ridge National Laboratory, Oak Ridge, TN 37831, USA*

[2] *Department of Physics, Southern University of Science and Technology, Shenzhen 518055, China*

[3] *Max Planck Institute for Chemical Physics of Solids, Nöthnitzer Str. 40, D-01187 Dresden, Germany*

[4] *Institut für Festkörper- und Materialphysik, Technische Universität Dresden, D-01069 Dresden, Germany*

[5] *Department of Physics and Astronomy, The University of Tennessee, Knoxville, TN 37996, USA*

[6] *Westlake Institute of Advanced Study, Hangzhou, 310024, P. R. China*

[7] *Theoretical Division, T-4 and CNLS, Los Alamos National Laboratory, Los Alamos, NM 87545, USA*

[8] *Shull-Wollan Center, Oak Ridge National Laboratory, Oak Ridge, TN 37831, USA*

[9] *Condensed Matter Physics and Materials Science Division, Brookhaven National Laboratory, Upton, NY 11973, USA*

[10] *Materials Science and Technology Division, Oak Ridge National Laboratory, Oak Ridge, TN 37831, U.S.A.*

[11] *Lviv Polytechnic National University, 79013 Lviv, Ukraine*

[12] *Neutron Technologies Division, Oak Ridge National Laboratory, Oak Ridge, TN 37831, USA*

Supplementary Note 1: Specific Heat and Phonon Correction

The temperature dependent specific heat C_p of $YbAlO_3$ is shown in Supplementary Fig. 1. A sharp peak anomaly is observed at $T_N = 0.88$ K in zero field, indicating a phase transition into the long-range antiferromagnetic (AFM) order at lower temperatures. By applying a magnetic field along the a-axis, the peak is gradually suppressed, and finally disappears in high fields. The lattice contribution of the phonons is estimated through the zero field specific heat of the nonmagnetic isostructural compound $YAlO_3$, shown as empty circles in Supplementary Fig. 1a. The magnetic specific heat in different fields is then extracted as $C_M = C_p - C_{phonon}$, as shown in Supplementary Fig. 1b, and all the magnetic specific heat data shown below and in the main text are extracted in the same way.

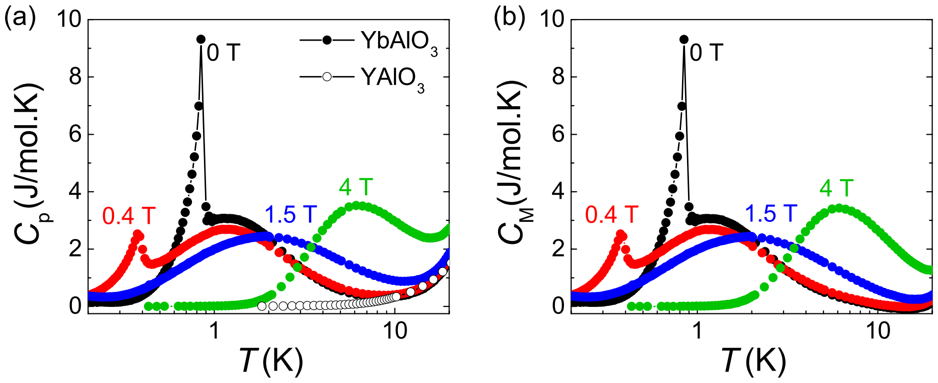

Supplementary Figure 1. Specific heat and phonon correction. (a) Temperature dependence of specific heat C_p of $YbAlO_3$ (solid circles) and $YAlO_3$ (empty circles), measured in different magnetic fields applied along the a-axis. (b) Magnetic specific heat C_M in different magnetic fields after the correction for the phonon contribution.

Supplementary Note 2: Two Magnetic Sublattices and Staggered Field

Supplementary Figure 2 illustrates the magnetic structure of $YbAlO_3$ in zero field. The Yb moments are confined in the ab-plane at $z = 1/4c$ (Supplementary Fig. 2a) and $z = 3/4c$ (Supplementary Fig. 2b). A static magnetic order is established in $YbAlO_3$ below $T_N = 0.88$ K, and the magnetic configuration of $AxGy$ is selected by the dipole-dipole interaction. As indicated by the red dashed lines, the calculated dipole-dipole interactions between near neighbors are $J_1 = -0.058$ K, $J_2 = -0.037$ K, $J_3 = -0.10$ K and $J_4 = 0.03$ K in the $z = 1/4c$ plane, assuming a static moment on each Yb site of 3.8 μ_B/Yb. The magnetic moments in $z = 3/4c$ are antiparallel to moments in $z = 1/4c$, and the magnetic interactions are of the

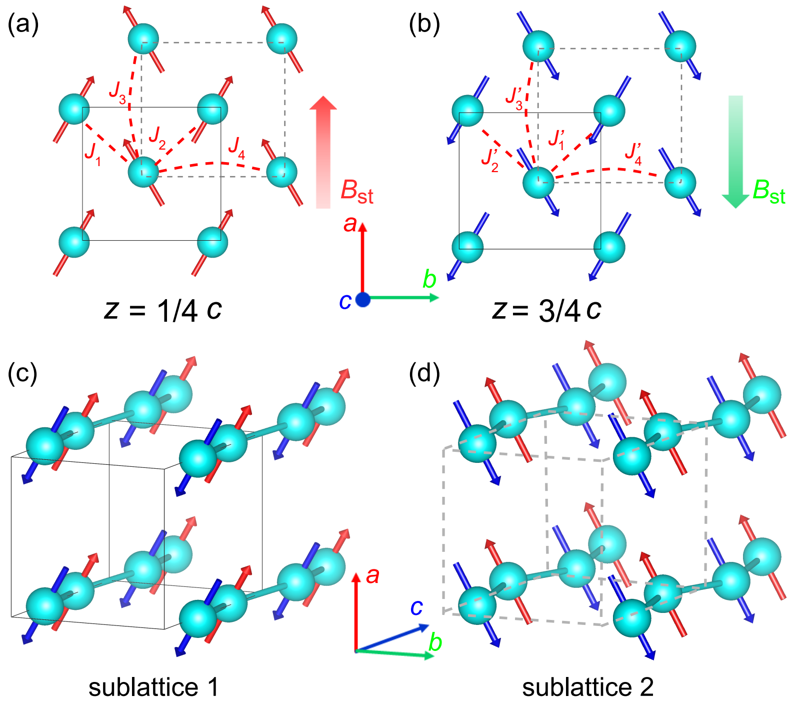

Supplementary Figure 2. Magnetic structure of $YbAlO_3$. (a)-(b) Magnetic Yb moment configurations in planes at $z = 1/4c$ (a) and $z = 3/4c$ (b), respectively. The black solid and gray dashed lines indicate the unit cell for two magnetic sublattices. The red dashed lines indicate the near neighbor dipole-dipole interaction between the Yb Ising like moments, and this interaction leads to a net staggered field $B_{\rm st}$, with opposite directions in $z = 1/4c$ and $z = 3/4c$ planes, as indicated by the big red (a) and green (b) arrows. (c)-(d) Two separated magnetic sublattices with one-dimensional Yb AFM chains along the c-axis, where the Yb moments have the same local Ising axis in each one magnetic sublattice.

same magnitude, but with opposite signs. In the ordered state, the molecular field produced by these interactions results into a staggered field $B_{\rm st} = -2\sum_{i=1}^{4} J_i \simeq 0.33$ K. Since the dipole iteration is long range, we have extended this calculation to about forty-eight near neighbors, and the staggered molecular field saturates to about 0.49 K, which is quite close to the best fit $B_{\rm st} = 0.27J \sim 0.66$ K used in the DMRG calculation in the main text. With the picture of two sublattices in a staggered field (Supplementary Fig. 2c,d), one can clearly see one dimensioanl (1D) Yb antiferromagnetic (AFM) chains along the c-axis emerging from the three dimensional perovskite crystal structure. This observation naturally leads to the 1D Hamiltonian (1) proposed in the main text.

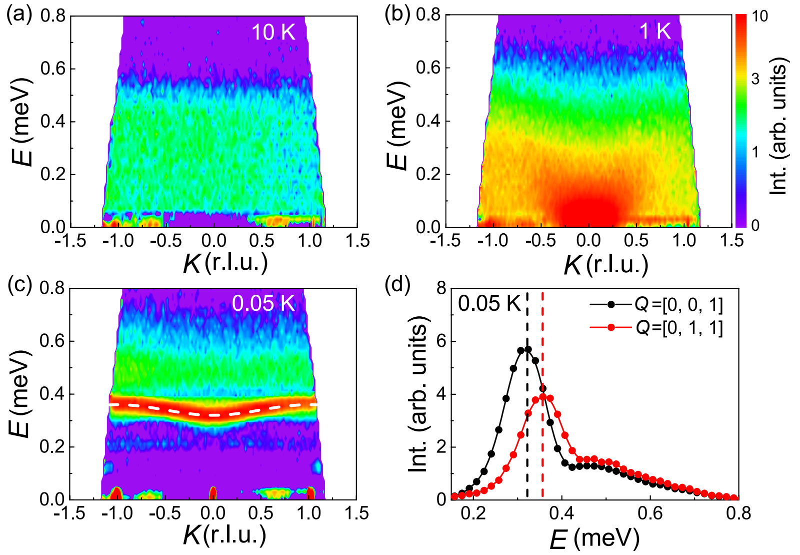

Supplementary Figure 3. INS spectra of $YbAlO_3$ along the wave vector $(0K1)$ direction. (a)-(c) Energy dependent INS spectrum integrated over wave vector $H = [-0.2, 0.2]$ r.l.u. and $L = [0.9, 1.1]$ r.l.u., measured in zero field and at different temperatures 10 K (a), 1 K (b), and 0.05 K (c), as indicated. The dashed line in (c) is described in the text. (d) Energy cuts measured at temperature 0.05 K, integrated over wave vector $H = [-0.2, 0.2]$ r.l.u., $L = [0.9, 1.1]$ r.l.u., $K = [0, 0.2]$ r.l.u. and $K = [0.9, 1.1]$ r.l.u., respectively. The vertical dashed lines indicate the peak positions of 0.32 meV at $\boldsymbol{Q} = (0, 0, 1)$ and 0.36 meV at $\boldsymbol{Q} = (0, 1, 1)$.

Supplementary Note 3: Estimation of interchain interaction

As expected from the two-magnetic-sublattice model, where one-dimensional spin chains run along the c-axis, we observe a spinon continuum along the $(00L)$ direction with only weak modulation in the direction perpendicular to the chain. Supplementary Figure 3a-c shows the inelastic neutron scattering (INS) spectrum of $YbAlO_3$ at different temperatures along the wave vector direction $(0K1)$. At $T \sim 10$ K, well above the magnetic phase transition, thermal fluctuations are large enough to destroy the interchain coupling in the ab-plane, and a broad flat continuum is observed (Supplementary Fig. 3a) with non-observable dispersion along the $(0K1)$ direction. At $T \sim 1$ K, i. e. just above the ordering transition, short-range correlations start to build up with broad diffuse scattering developing around $\boldsymbol{Q} = (0, 0, 1)$, and a weak variation is observed in the spectrum (Supplementary Fig. 3b). Static (ordered) magnetic moments emerge upon further lowering the temperature down to 0.05 K, which is deep inside the AFM order, and the resulting effective staggered field opens a gap at the

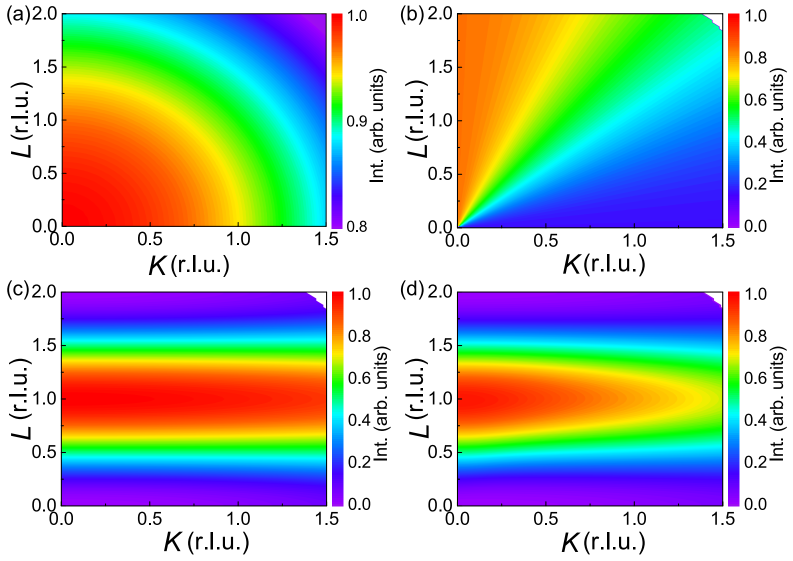

Supplementary Figure 4. Calculated magnetic neutron scattering factor in the $(0KL)$ scattering plane. (a) Contour plot of the magnetic form factor ($|F(\boldsymbol{Q})|^2$) of Yb^{3+}. (b) Contour plot of the polarization factor $(1-\hat{Q}_z\hat{Q}_z)$, with the magnetic Ising moments constricted at an angle $\varphi = \pm 23.5°$ with the a-axis. (c) Contour plot of the antiferromagnetic static spin structure factor ($S(\boldsymbol{Q})$) for nearest neighbor exchange along the c-axis. (d) Contour plot of the overall cross section (after including the magnetic form factor) (a), polarization factor (b), and the AFM static structure factor (c).

AFM wave vector $\boldsymbol{Q} = (0, 0, 1)$ (Supplementary Fig. 3c). At this temperature, the lowest peak of the dispersion along $(0K1)$ can be phenomenologically described as

$$E_\mathrm{k} = \sqrt{\left[2J'\left(1 - \cos k\pi\right)\right]^2 + E_0^2}, \tag{1}$$

where J' is the interchain coupling and E_0 is the gap opened by the staggered field at $L = 1$. Energy cuts taken at $\boldsymbol{Q} = (0, 0, 1)$ and $(0, 1, 1)$ are shown in Supplementary Fig. 3d. With fitting parameters $J' = 0.04$ meV, and $E_0 = 0.32$ meV, the above dispersion relation reproduces the peak positions: 0.32 meV at $(0, 0, 1)$ and 0.36 meV at $(0, 1, 1)$. This analysis indicates that the effective interchain interaction is roughly 20% of the intrachain exchange: $J'/J \simeq 0.04/0.21 \simeq 0.19$.

Supplementary Note 4: Polarization Factor and Longitudinal Fluctuations

DC magnetization and CEF studies suggest well separated doublets for the Yb^{3+} ion in $YbAlO_3$. Since the first exited CEF levels are high (29 meV $\sim$ 345 K), the low temperature

magnetic properties are dominated by the ground state doublets, which can be effectively described as pseudo-spin S=1/2 states with anisotropic g-tensors:

$$M_z = M_\mathrm{s} = 3.8\mu_\mathrm{B}/\mathrm{Yb}, \qquad g_\mathrm{eff}^{zz} = 7.6, \tag{2}$$

$$M_{xy} = M_\mathrm{c} = 0.23\mu_\mathrm{B}/\mathrm{Yb}, \qquad g_\mathrm{eff}^{xx} = g_\mathrm{eff}^{yy} = 0.46, \tag{3}$$

where z is chosen along the local moment easy axis. This Ising-like g-tensor anisotropy does not necessarily lead to anisotropic interactions, but it manifests in the magnetic neutron scattering cross-section [1, 2]:

$$\frac{\mathrm{d}^2\sigma}{\mathrm{d}\Omega\mathrm{d}E} \propto |F(\boldsymbol{Q})|^2 \sum_{\alpha\beta} (\delta_{\alpha\beta} - \hat{Q}_\alpha \hat{Q}_\beta)(g^{\alpha\beta})^2 S^{\alpha\beta}(\boldsymbol{Q}, E). \tag{4}$$

Here $|F(\boldsymbol{Q})|^2$ is the magnetic form factor of Yb^{3+}, $\delta_{\alpha\beta} - \hat{Q}_\alpha \hat{Q}_\beta$ is the polarization factor, and $S^{\alpha\beta}(\boldsymbol{Q}, E)$ is the dynamical spin structure factor of different components. Since $(g^{zz})^2/(g^{xx})^2 \simeq 273$, transverse contributions to the total magnetic scattering can be neglected and the overall magnetic scattering is dominated by longitudinal fluctuations:

$$\frac{\mathrm{d}^2\sigma}{\mathrm{d}\Omega\mathrm{d}E} \propto |F(\boldsymbol{Q})|^2 (1 - \hat{Q}_z \hat{Q}_z)(g^{zz})^2 S^{zz}(\boldsymbol{Q}, E) + O\left((g^{xx})^2\right) + O\left((g^{yy})^2\right). \tag{5}$$

The magnetic polarization factor can be obtained from the angle between the Yb moments and the a-axis ($\varphi = \pm 23.5^\circ$):

$$1 - \hat{Q}_z \hat{Q}_z = 1 - \frac{Q_k^2 \sin^2\varphi}{Q_k^2 + Q_l^2} = \frac{Q_k^2 \cos^2\varphi + \mathrm{Q}_\mathrm{l}^2}{Q_k^2 + Q_l^2}. \tag{6}$$

Assuming AFM interactions between nearest-neighbor Yb ions along the c-axis, we can also obtain the static magnetic structure factor

$$S(\boldsymbol{Q}) = \int S(\boldsymbol{Q}, E)\, \mathrm{d}E = |\sum_\mathrm{i} \boldsymbol{m}_\mathrm{i} e^{-i\boldsymbol{Q}\cdot\boldsymbol{r}_\mathrm{i}}|^2$$
$$= 2|m|^2 \cdot \left[\sin^2 \frac{\pi(l + 0.12k)}{2} + \sin^2 \frac{\pi(l - 0.12k)}{2}\right], \tag{7}$$

where $\boldsymbol{m}_\mathrm{i}$ are the Yb magnetic moments located at positions $\mathbf{r}_\mathrm{i}$ [3]. The Yb atoms form into a zig-zag chain along the crystal c-axis in the orthorhombic perovskite structure, with small distortions in a and b directions (Supplementary Fig. 2). The distortion angle along the a-axis is about $\sim 2.2^\circ$, which is negligible for scattering in the $(0KL)$ plane. The larger distortion angle $\sim 9.9^\circ$ along the b-aixs introduces an additional wave vector dependence

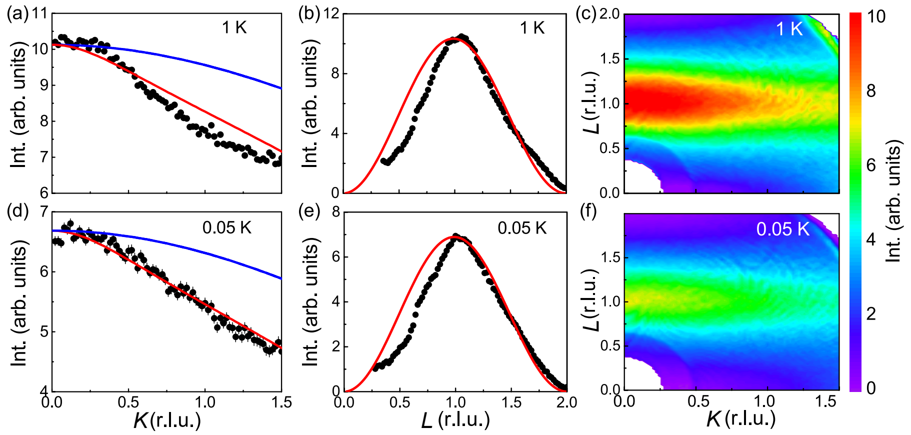

Supplementary Figure 5. Experimental magnetic INS spectrum of $YbAlO_3$ in the $(0KL)$ plane. (a),(d) Constant energy cut integrated over $E = [0.1, 0.8]$ meV, and wave vector $H = [-0.2, 0.2]$ r.l.u., $L = [0.9, 1.1]$ r.l.u., measured at temperatures 1 K (a), and 0.05 K (d), respectively. The blue lines are the calculated form factor ($|F(\boldsymbol{Q})|^2$) only, and the red lines are the product of the calculated form and polarization factors. (b),(e) Constant energy cut integrated over energy $E = [0.1, 0.8]$ meV, and wave vector $H = [-0.2, 0.2]$ r.l.u., $K = [-0.2, 0.2]$ r.l.u., measured at temperatures 1 K (d), and 0.05 K (e), respectively. The red lines are the calculated magnetic structure factor. (c),(f) Contour plots of the INS spectrum in the $(0KL)$ scattering plane, integrated over energy $E = [0.1, 0.8]$ meV, at temperatures 1 K (c), and 0.05 K (f), respectively.

with $l \pm 0.12k$ in the scattering factor, making the spectral weight around $L = 1$ more spread at higher values of wave vector K.

The calculated magnetic form factor ($|F(\boldsymbol{Q})|^2$), polarization factor ($1 - \hat{Q}_z\hat{Q}_z$) and AF static spin structure factor ($S(\boldsymbol{Q})$) are shown in Supplementary Fig. 4a-c. The overall magnetic scattering factor is plotted in Supplementary Fig. 4d. The calculated wave vector dependence of the magnetic scattering can be directly compared to the experimental results, as presented in Supplementary Fig. 5.

The wave vector dependence along the $(0K1)$ direction, measured at temperatures 1 K and 0.05 K, is shown in Supplementary Fig. 5a,d, respectively. The blue lines are the calculated form factor ($|F(\boldsymbol{Q})|^2$), which changes about 10% along the K direction, while the additional 20% changes are well captured when the polarization factor is included (red lines). The polarization factor is a constant for $K = 0$ and the intensity variation along the $(00L)$ direction is dominated by the spin structure factor $S(\boldsymbol{Q})$. As shown in Supple-

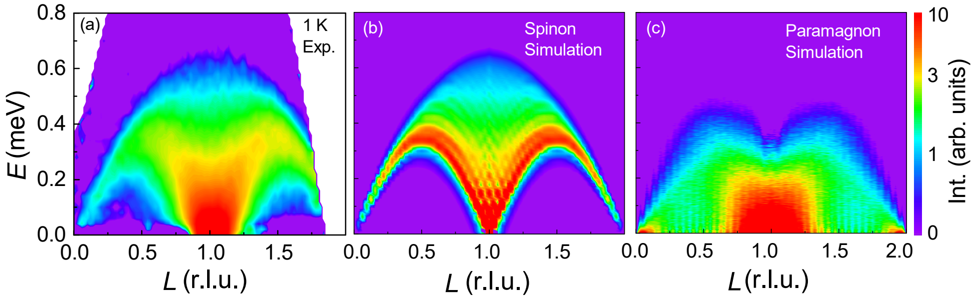

Supplementary Figure 6. Comparison of the spin excitation spectra. (a) Experimental INS spectrum at 1 K (the experimental Néel temperature is $T_N \approx 0.88$ K). (b) Longitudinal spin structure factor $S^{zz}(\boldsymbol{Q}, E)$ of model Eq. (2) in the main text, computed by DMRG at $T = 0$. (c) Longitudinal spin structure factor $S^{zz}(\boldsymbol{Q}, E)$ of the classical spin model (8) at 0.5 K (the corresponding Néel temperature is $T_N \approx 0.45$ K), obtained from the Landau-Lifshitz dynamics (the energy E has been rescaled by $\sqrt{3}/2$ to account for the size of $S = 1/2$).

mentary Fig. 5b,e, both the L-dependence below and above the AFM phase transition are well described by the calculated magnetic structure factor (red lines). The experimental INS spectrum integrated over $E = [0.1, 0.8]$ meV in the $(0KL)$ scattering plane is shown in Supplementary Fig. 5c,f, where an overall consistency is observed with the calculated pattern shown in Supplementary Fig. 4d. This demonstrates that the magnetic scattering is dominated by longitudinal fluctuations along the local easy-axis of the Yb moments.

Supplementary Note 5: Comparison of paramagnon and two-spinon continuum

In the main text, we have shown that the magnetic excitations of $YbAlO_3$ have a two-spinon continuum at $T = 1$ K (Fig. 3a), right above the Néel temperature T_N. The spectrum is consistent with our $T = 0$ DMRG calculation (Fig. 3c). Furthermore, the broadening of the experimental data can also be captured by a finite temperature tDMRG calculation [4].

While everything is consistent with the two-spinon continuum, it is still interesting to ask if one can obtain the same excitation spectrum from the paramagnon picture. For this comparison, we use the 3-dimensional version of the effective model, with classical spins:

$$\mathcal{H} = J \sum_{\langle ij \rangle} \boldsymbol{S}_i \cdot \boldsymbol{S}_j + J_{\text{inter}} \sum_{\langle ij \rangle'} S_i^z S_j^z, \tag{8}$$

where $\langle ij \rangle$ denotes the nearest neighbor bonds along the chain, and $\langle ij \rangle'$ denotes the nearest neighbor bonds in between chains. Following the best fit from the main text, we use $J =$

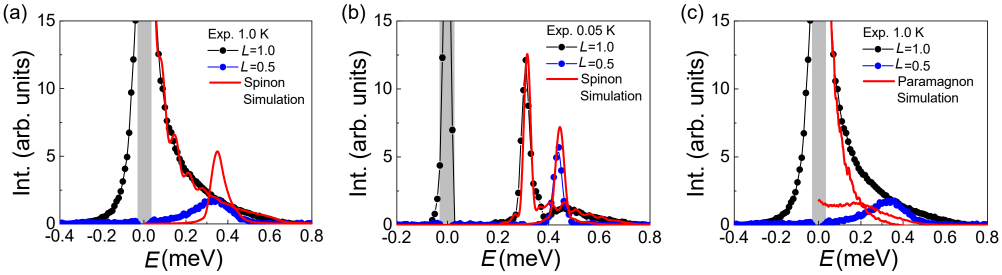

Supplementary Figure 7. Energy cuts of the spin excitation spectrum. (a) Energy cut at wave vector $H = [-0.2, 0.2]$ r.l.u., $K = [-1, 1]$ r.l.u., $L = [0.9, 1.1]$ r.l.u., and $L = [0.4, 0.6]$ r.l.u. at 1.0 K, and the red line is the energy cut of $S^{zz}(\boldsymbol{Q}, E)$ from $T = 0$ DMRG calculation with zero staggered field $B_{\rm st} = 0$. (b) Energy cut at wave vector $H = [-0.2, 0.2]$ r.l.u., $K = [-1, 1]$ r.l.u., $L = [0.9, 1.1]$ r.l.u., and $L = [0.4, 0.6]$ r.l.u. at 0.05 K, and the red line is the energy cut of the $T = 0$ DMRG calculation with staggered field $B_{\rm st}/J = 0.27$. In both (a) and (b), the simulated intensity are scaled by the same factor to match the experimental data (the artificial oscillations in the simulated curve are from the finite size effect). (c) Energy cut at wave vector $H = [-0.2, 0.2]$ r.l.u., $K = [-1, 1]$ r.l.u., $L = [0.9, 1.1]$ r.l.u., and $L = [0.4, 0.6]$ r.l.u. at 1.0 K, and the red line is the energy cut of $S^{zz}(\boldsymbol{Q}, E)$ from Landau-Lifshitz spin dynamics at $T = 0.5$ K. The vertical gray bar indicates the instrumental resolution, which is much narrower than the experimental spinon continuum.

0.21 meV, $J_{\rm inter} \approx \frac{B_{\rm st}}{2} \approx 0.028$ meV.

To calculate the excitation spectrum of the classical spin model (8), we first use Monte-Carlo simulation with Metropolis update to obtain the spin configurations in thermal equilibrium, on a $6\sqrt{2} \times 6\sqrt{2} \times 40$ lattice (40 being along the chain direction). After achieving thermalization from completely disordered spin configurations, we time-evolve the spins with Landau-Lifshitz dynamics, using in total 3×10^4 dynamical steps. The longitudinal component of structure factor is obtained by Fourier transformation of the spin correlation function $\langle S^z(\boldsymbol{r}_i, 0) S^z(\boldsymbol{r}_j, t)\rangle$, averaged over 32 independent replicas.

Supplementary Figures 6 and 7 show the comparison between the experimental INS spectrum and theory. While all of them show broad continuum centered around $\boldsymbol{Q} = (0, 0, 1)$, the details are clearly different: away from $\boldsymbol{Q} = (0, 0, 1)$, the DMRG calculation captures correctly the coherent excitations (Supplementary Fig. 7(a),(b)), while the Landau-Lifshitz simulation produces diffuse-like scattering at low energy (Supplementary Fig. 7(c)), i.e. a

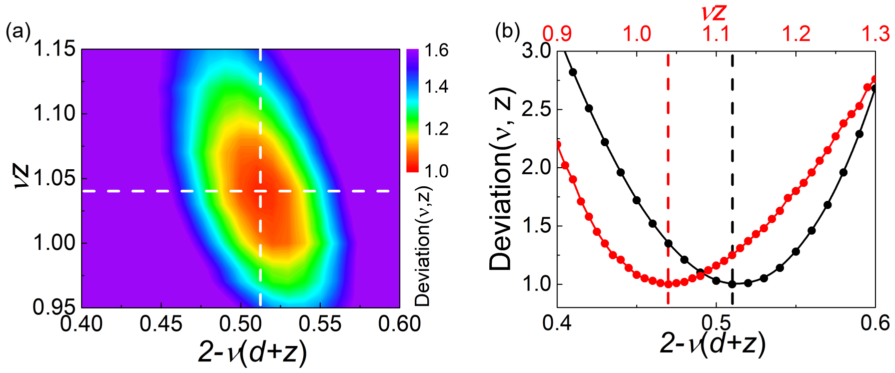

Supplementary Figure 8. Quantum critical scaling of $YbAlO_3$. (a) Contour plot of the calculated deviation from scaling as functions of νz and $2-\nu(d+z)$, using the magnetization data (see Fig. 7a-b in the main text). The white dashed lines indicate the global minimum at $\nu z = 1.04$ and $2-\nu(d+z) = 0.51$. (b) Calculated deviations for different scaling parameters around the minimum position.

simple paramagnon picture does not apply when the temperature is right above T_{N}.

Supplementary Note 6: Quantum critical scaling

The field dependence of the magnetization is presented in Fig. 7a in the main text. Following Ref. 5, we define the deviation from scaling as:

$$\mathrm{Deviation}(\nu, z) = \sum_{i,j} \sum_{i',j'} [\frac{\mathrm{d}M}{\mathrm{d}B}(T_{\mathrm{i}}, B_{\mathrm{j}}) - \frac{\mathrm{d}M}{\mathrm{d}B}(T_{\mathrm{i'}}, B_{\mathrm{j'}})]^2. \tag{9}$$

As shown in Supplementary Fig. 8a, the minimum deviation is reached for

$$\nu z = 1.04 \tag{10}$$

$$2 - \nu(d+z) = 0.51, \tag{11}$$

which is consistent with a free fermion fixed point: $\nu = 1/2$, $z = 2$ and $d = 1$.

SUPPLEMENTARY REFERENCES

[1] Mourigal, M. et al. Block Magnetic Excitations in the Orbitally Selective Mott Insulator $BaFe_2Se_3$. *Phys. Rev. Lett.* **115**, 047401 (2015).

[2] Wu, L. S. et al. Orbital-exchange and fractional quantum number excitations in an f-electron metal, Yb_2Pt_2Pb. *Science* **352**, 1206-1210 (2016).

[3] Zaliznyak, I. et al. Spin-liquid polymorphism in a correlated electron system on the threshold of superconductivity. *Proc. Natl. Acad. Sci. U.S.A.* **84**, 10316 (2015).

[4] Lake, B. et al. Multispinon Continua at Zero and Finite Temperature in a Near-Ideal Heisenberg Chain. *Phys. Rev. Lett.* **111**, 137205 (2013).

[5] Yang, B. et al. Quantum criticality and the Tomonaga-Luttinger liquid in one-dimensional Bose gases. *Phys. Rev. Lett.* **119**, 165701 (2017).
