# Supplementary material for: Tomonaga–Luttinger liquid behavior and spinon confinement in YbAlO3
Source: Nat Commun. 2019 Feb 11;10:698. doi: 10.1038/s41467-019-08485-7 (PMC6370837; doi:10.1038/s41467-019-08485-7)
Supplement: Supplementary file 2 — Peer Review File [file 41467_2019_8485_MOESM2_ESM.pdf]

Reviewers' comments:

Reviewer #1 (Remarks to the Author):

The work presents the detailed experimental study of magnetic excitations in insulating compound $YbAlO_3$. According to the abstract there are two major messages of the work
(i) Tomonaga-Luttinger behaviour,
(ii) deconfinement of spinons.
This is a very detailed study. It includes elastic and inelastic neutron scattering (INS) for different temperatures and magnetic fields as well as magnetization and magnetic specific heat measurements. On the theoretical side the analysis is supported by DMRG simulations for a 1D Heisenberg chain with specific parameters.

While the presented data are interesting and useful, unfortunately I do not see qualitatively new results.
The Tomonaga-Luttinger behaviour has been discussed and observed before, so this point is not new.
A detailed study of confinement/deconfinement transition could be a novel point. Such a study should include an analysis of evolution of the confinement length with temperature, the energy scale of the confinement, spinon-spinon scattering mean free path in the deconfined phase and other related issues. However, the work just presents INS spectra well below T_N (confined phase) and just above T_N ("deconfined" phase), Fig.2a. I am even not convinced that Fig2a(i) proves deconfinement. What is the ratio of the spinon thermal wave length \lambda_T and the spinon mean free path l? Does it make sense to talk about deconfinement if \lambda_T ~ l? Purely phenomenologicaly a description in terms of broad paramagnons seems applicable too. I am not sure that DMRG can shed light on this issue.

All in all I have no doubts in the presented results, they are interesting and important. Nevertheless, I cannot recommend the present version of the manuscript for publication in Nature communications. In my opinion a specialized journal is more appropriate.

Reviewer #2 (Remarks to the Author):

This manuscript reports the results of inelastic neutron scattering on antiferromagnetic linear chain system $YbAlO_3$, in which magnetic Yb^{3+} has large easy-axis anisotropy. The authors found that magnetic excitations at zero and finite magnetic fields are well described by isotropic exchange, Zeeman tern by applied uniform magnetic field and staggered magnetic field induced parallel to the applied magnetic field by dipole interaction from the neighboring chains below TN=0.88 K, which confine spinons. The results are very interesting. However, I have questions and comments as follows:
1. The authors state that isotropic Yb^{3+} observed in $YbAlO_3$ is rare case. However, isotropic Yb^{3+} forming antiferromagnetic chain was already found in Yb_4As_3, [M. Kohgi et al., Phys. Rev. Lett. 86, 2439 (2001)]. In Yb_4As_3, the staggered field is induced perpendicular to the applied magnetic field. Consequently, Yb_4As_3 is described by quantum sine-Gordon model. The authors should cite this paper.

2. Magnetic excitations are described by the model expressed by eq. (1). Is magnetization process shown in Fig. 4(a) explainable by this model? Are the magnetic parameters evaluated from the analysis of the magnetization process consistent with the model of eq. (1)?
3. In magnetization process shown in Fig. 4(a), 1/3-plateau and a jump at one-half of saturation magnetization are observed. Can authors explain these anomalies?

Reviewer #3 (Remarks to the Author):

The authors report experimental results on $YbAlO_3$ with neutron scattering and thermodynamic measurements. They analyzed the results by means of ad hoc DMRG calculations on the Heisenberg spin-1/2 chain under a uniform and staggered magnetic fields. From the fitting of inelastic neutron scattering spectra and scaling behaviors of the magnetization and the specific heat, they claim that the compound hosts antiferromagnetic Heisenberg spin chains coupled to each other by the magnetic dipole-dipole interactions and display Tomonaga-Luttinger liquid behaviors in the unordered phase and the confinement in the ordered phase.
The experimental results reported in the manuscript are of high quality. The fittings are also reasonable. So the results suggest a possible rare example of rare-earth Heisenberg spin chains from rare-earth elements.

I will recommend the publication of this manuscript if the following questions are answered and criticisms are met.

1. Since one of key experimental observations is the Tomonaga-Luttiner liquid, the authors should cite the original papers by S. Tomonaga and by J.M. Luttinger. Furthermore, a choice of citations of some papers does not necessarily respect the original papers. This could be modified.

2. The organization of this manuscript is not very nice. Some key results/analyses/discussions are included in the supplement. Since the manuscript is submitted to Nature Communications which has no page limit, it is desirable to include significant parts of the supplement into the main text. For instance, Fig.S2 and its explanation play a crucial role in establishing and explaining the easy-axis magnetic anisotropy. I also don't understand why Fig.S8 summarizing the phase diagram is included in the supplement but not in the main text. A similar phase diagram has already been included as Fig.5c, but it is based on the magnetic specific heat. Another from the inelastic neutron scattering intensity should be compared directly without taking a look at the supplement. Similarly, scaling plots (Fig.5) provide one of the important results of the manuscript. This could also be included in the main text.

3. The authors claim that that the theoretical analyses of their experimental results indicate that the Yb spins are modeled by Eq.(1). For instance, they summarize, “Both, the gapless spinon spectrum and the critical behavior around the saturation field at T>T_N unambiguously demonstrate that the intra-chain exchange is Heisenberg like to a very good approximation.” On the contrary to this claim, the results just provide circumstantial evidence but not compelling. For instance, the level of exchange magnetic anisotropy could be analyzed from the shape of the wavevector-dependent neutron scattering spectra. It could also be tested with NMR and ESR experiments.
It is more or less clear that the scaling behavior in the case of B>B_s should approach the free-fermion, since such a strong magnetic field as fully polarizes the spin reduces the symmetry to U(1) at most. It would be possible to extract a change in the Tomonaga-Luttinger parameter in the scaling analysis with changing magnetic field. This analysis is theoretically solid and will appreciably strengthen the case.
Also from a theoretical point of view, it would be possible to estimate the form of exchange interactions, as it has been performed for $Yb_2Ti_2O_7$ (K.A. Ross et al., PRX 1, 021002 (2011); L.-J. Chang et al., Nature Comms. 3:992 (2012)) in S. Onoda, J. Phys.: Conf. Series 320, 012065 (2011).

Any of these attempts is rather important as the authors admit that a Heisenberg interaction between Yb magnetic moments is a surprise. It is particularly surprising when the exchange interactions have spatial anisotropy and the g-tensor is anisotropic.
The authors could choose whether they add such direct evidence to strengthen the case or reword the associated statements more carefully by commenting on the other possibilities.

4. There are several misleading phrases and sentences:
p.5.
The phrase "the spin gap closes abruptly within a narrow temperature range of about T_N\pm0.1 K" does not make sense, since the spin gap, if it exists, means that the ground state is spin-singlet. A similar expression also appears in the Fig.3 caption. The "spin gap" should read the gap in spin exciations or the magnon/spin wave gap.

p.7.
“between intrachain and intrachain interactions” is clearly odd.

Response to the Referees and Changes Made in the Manuscript

The list of answers to the Referees is given below.
Referee questions, remarks or comments are in black, our replies are in red.

New text and changes in the manuscript are highlighted by blue color.

Reply to Referee #1

The work presents the detailed experimental study of magnetic excitations in insulating compound YbAlO3. According to the abstract there are two major messages of the work (i) Tomonaga-Luttinger behaviour, (ii) deconfinement of spinons. This is a very detailed study. It includes elastic and inelastic neutron scattering (INS) for different temperatures and magnetic fields as well as magnetization and magnetic specific heat measurements. On the theoretical side the analysis is supported by DMRG simulations for a 1D Heisenberg chain with specific parameters.

We thank the referee for the positive comments about the manuscript.

While the presented data are interesting and useful, unfortunately I do not see qualitatively new results.
The Tomonaga-Luttinger behaviour has been discussed and observed before, so this point is not new.
A detailed study of confinement/deconfinement transition could be a novel point. Such a study should include an analysis of evolution of the confinement length with temperature, the energy scale of the confinement, spinon-spinon scattering mean free path in the deconfined phase and other related issues. However, the work just presents INS spectra well below T_N (confined phase) and just above T_N
("deconfined" phase), Fig.2a. I am even not convinced that Fig2a(i) proves deconfinement. What is the ratio of the spinon thermal wave length \lambda_T and the spinon mean free path l? Does it make sense to talk about deconfinement if \lambda_T ~ l? Purely phenomenologically a description in
terms of broad paramagnons seems applicable too. I am not sure that DMRG can shed light on this issue.
All in all I have no doubts in the presented results, they are interesting and important. Nevertheless, I cannot recommend the present version of the manuscript for publication in Nature communications. In my opinion a specialized journal is more appropriate.

As the referee indicates, the confinement/deconfinement transition is one of the novel points of this manuscript. As we explain in the conclusions, the realization of Heisenberg chains coupled by Ising interchain interactions is another novel point. Following the referee suggestion, we have included an analysis of the deconfinement length in the new version of the manuscript. The spinon thermal

wavelength right above the Neel temperature can be extracted from Figure 3 of the manuscript. Given that the Neel temperature is a fraction of a Kelvin (<0.1 meV), the spinon wavelength is of order 10 lattice spaces. However, this is not the length scale that must be compared against the spinon mean free path. The relevant length scale is the confinement radius, whose staggered field and temperature dependence are given in the new version of the manuscript. The confinement radius ξ_c is given by the inverse gap, $1/\Delta$, which goes to zero at $T=T_N$. Given that the gap for spin excitations is proportional to the inverse correlation length, $\Delta \propto 1/\xi$, near T_N, the confinement radius is proportional to the magnetic correlation length, whose temperature dependence is controlled by the exponent ν: $\xi_c \propto \xi \propto (T_N-T)^{-\nu}$, with $\nu \cong 0.63$ for the 3D Ising model. The measured INS spectrum shows that the gap goes to zero at the Neel temperature. In addition, the comparison shown in Fig. 3 shows that the INS spectrum right above the Neel temperature is still well described by the T=0 DMRG calculation. The agreement becomes better right above the IC-AFM phase (see Fig. 4) because the Neel temperature is significantly suppressed above B_c=0.35T.

To demonstrate that a phenomenological description based on broad paramagnons does not describe the observed dynamical spin structure factor, we simulated a classical version of the 3D spin model and computed the dynamical spin structure factor (Landau-Lifshitz spin dynamics combined with Monte Carlo sampling for thermal equilibration). The result of this classical simulation is presented in the right panel of the figure below this text (more details explained in the updated supplemental material).

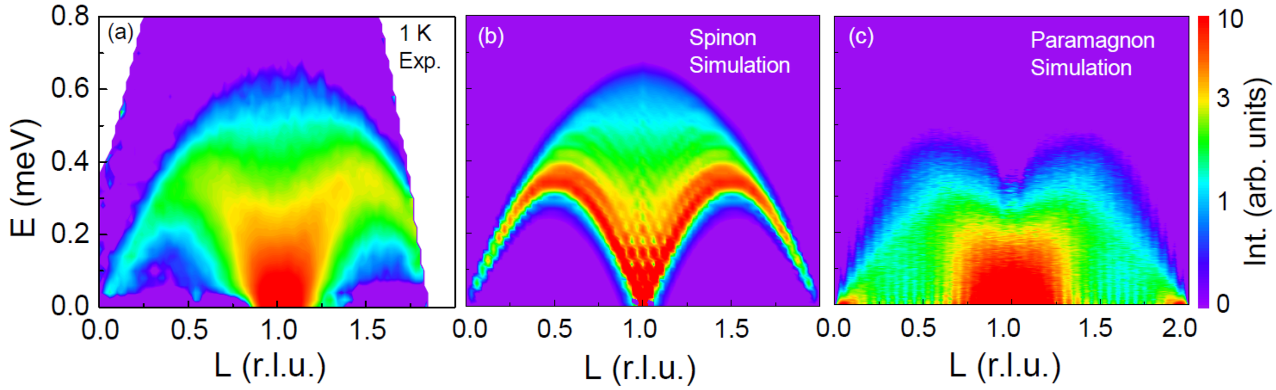

The left panel (a) is the measured $S(q,\omega)$ right above the Neel temperature; the middle panel (b) shows the results obtained with the single-chain Hamiltonian given in Eq. (2) of the manuscript; and the right panel (c) shows the results obtained from the 3D classical spin model with Landau-Lifshitz dynamics. We note that the temperature enters only through the magnitude of the staggered molecular field in the DMRG calculation. In other words, the DMRG result in panel (b) was obtained by setting the staggered field *and the temperature* to zero. A more realistic DMRG calculation should also include the effect of the finite temperature. Incidentally, this DMRG calculation has been done in the paper by B. Lake et al., PRL 111, 137205 (2017) (shown in Fig.5b therein). The result is reproduced below:

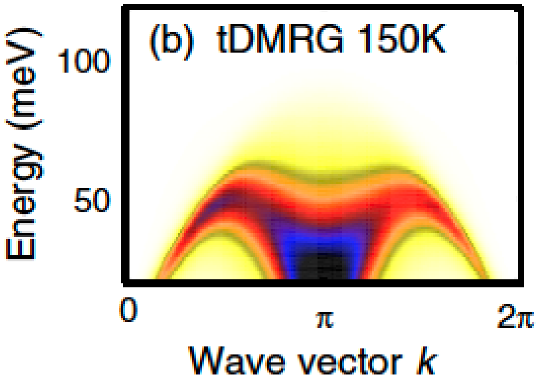

It is clear that this figure, or even the T=0 calculation shown in panel (b) of the above figure, reproduce very well the measured S(q,ω). In contrast, the result obtained by a phenomenological description (paramagnons) does not reproduce the spectral distribution that is observed in the experiment.

In summary, the observed suppression of the gap (inverse of confinement radius) at a temperature that is low enough to still observe the characteristic two-spinon continuum of the T=0 spectrum is the requirement for observing a confinement-deconfinement transition. The agreement between the experimental data and a theory that describes this confinement-deconfinement transition is the main piece of evidence. Further evidence is provided by the fact that a phenomenological (classical) description fails to reproduce the experimental data. We thank the referee for bringing up this important point and giving us the opportunity of providing further evidence of deconfinement.

Reply to Referee #2

This manuscript reports the results of inelastic neutron scattering on antiferromagnetic linear chain system YbAlO3, in which magnetic Yb3+ has large easy-axis anisotropy. The authors found that magnetic excitations at zero and finite magnetic fields are well described by isotropic exchange, Zeeman tern by applied uniform magnetic field and staggered magnetic field induced parallel to the applied magnetic field by dipole interaction from the neighboring chains below TN=0.88 K, which confine spinons. The results are very interesting. However, I have questions and comments as follows:

We thank the referee for the positive comments about the manuscript.

1. The authors state that isotropic Yb3+ observed in YbAlO3 is rare case. However, isotropic Yb3+ forming antiferromagnetic chain was already found in Yb4As3, [M. Kohgi et al., Phys. Rev. Lett. 86, 2439 (2001)]. In Yb4As3, the staggered field is induced perpendicular to the applied magnetic

field. Consequently, Yb4As3 is described by quantum sine-Gordon model. The authors should cite this paper.

We thank Referee 2 for pointing out the references on Yb4As3. We have included a discussion on Yb_4As_3 and cited the above-mentioned papers in the updated version of the manuscript.

2. Magnetic excitations are described by the model expressed by eq. (1). Is magnetization process shown in Fig. 4(a) explainable by this model? Are the magnetic parameters evaluated from the analysis of the magnetization process consistent with the model of eq. (1)?

Before answering this question, we point out that Eq. (1) in the old version of the manuscript became Eq. (2) in the new version.

While the field dependence of the magnetization can in principle be described with an effective one-dimensional model, Eq. (2) must be generalized in the following sense. At low fields ($B<B_c$), the molecular inter-chain field is simply the staggered field B_{st} that appears in the second term of Eq. (2). Above B_c, the magnetic ordering along the chain direction is no longer a simple two-sublattice antiferromagnetic ordering. The inter-chain molecular field must then be computed self-consistently, implying that for each field it is necessary to do multiple iterations of DMRG calculation until achieving convergence of the inter-chain molecular field. This tedious calculation is beyond the scope of this manuscript. We note in passing that this is an important difference relative to Yb4As3, where the staggered magnetic field is induced by a DM interaction instead of being generated by the inter-chain exchange coupling.

3. In magnetization process shown in Fig. 4(a), 1/3-plateau and a jump at one-half of saturation magnetization are observed. Can authors explain these anomalies?

In the manuscript, we are providing a qualitative argument that explains why the 1/3-plateau is favored (the ↑↑↓ state is the only magnetic ordering between B_c and B_{sat} that simultaneously minimizes the intra-chain Heisenberg exchance and inter-chain Ising interactions). We note that the Heisenberg exchange favors a $\pm 2k_f$ magnetic ordering, while the Ising interactions favor collinear ordering. Such collinear ordering is only compatible with $2k_f = 0, \pi, \pm 2\pi/3$. $2k_f = \pi$ corresponds to the magnetic ordering for $B<B_c$. $2k_f = 2\pi$ corresponds to the fully saturated phase for $B=B_{sat}$. $2k_f = \pm 2\pi/3$ corresponds to the observed plateau phase at $M/M_{sat}=1/3$.

The magnetization data shows a higher slope (not a jump) at $M/M_{sat}=1/2$. While the origin of this anomaly is beyond the scope of this manuscript, a possible scenario is that it is produced by a commensurate collinear ordering ↑↑↑↓. We note that this potential ordering includes $q = \pi$ component, in addition to the field induced $q = 0$ component and the $q = \pm 2k_f$ with $2k_f = \pi/2$. Consequently, we do expect an appreciable magnetization plateau for this magnetization value. However, we still expect the square root behavior of the magnetization at a commensurate-incommensurate transition ($h = h_c$) in d=1+1: $m \sim \sqrt{h - h_c}$. This behavior, which is ultimately

induced by the fermion character of the underlying particles (S=1/2 spins become spinless fermions in D=1+1), leads to a divergence of dm/dh at $h = h_c$. Given that this is a speculative argument, we rather not including these arguments in the present manuscript and wait until having a more quantitative theory of the evolution of the magnetic ordering as a function of magnetic field.

Reply to Referee #3

The authors report experimental results on YbAlO3 with neutron scattering and thermodynamic measurements. They analyzed the results by means of ad hoc DMRG calculations on the Heisenberg spin-1/2 chain under a uniform and staggered magnetic fields. From the fitting of inelastic neutron scattering spectra and scaling behaviors of the magnetization and the specific heat, they claim that the compound hosts antiferromagnetic Heisenberg spin chains coupled to each other by the magnetic dipole-dipole interactions and display Tomonaga-Luttinger liquid behaviors in the unordered phase and the confinement in the ordered phase. The experimental results reported in the manuscript are of high quality. The fittings are also reasonable. So, the results suggest a possible rare example of rare-earth Heisenberg spin chains from rare-earth elements.

We thank the referee for the positive comments about the manuscript.

I will recommend the publication of this manuscript if the following questions are answered and criticisms are met.

1. Since one of key experimental observations is the Tomonaga-Luttiner liquid, the authors should cite the original papers by S. Tomonaga and by J.M. Luttinger. Furthermore, a choice of citations of some papers does not necessarily respect the original papers. This could be modified.

We have added the reference in the updated version of the manuscript. We have also revised the other references to cite the original papers. The new version of the manuscript contains multiple new references.

2. The organization of this manuscript is not very nice. Some key results/analyses/discussions are included in the supplement. Since the manuscript is submitted to Nature Communications which has no page limit, it is desirable to include significant parts of the supplement into the main text. For instance, Fig.S2 and its explanation play a crucial role in establishing and explaining the easy-axis magnetic anisotropy.

We have reorganized and enlarged the manuscript following the suggestion of the referee.

I also don't understand why Fig.S8 summarizing the phase diagram is included in the supplement but not in the main text. A similar phase diagram has already been included as Fig.5c, but it is

based on the magnetic specific heat. Another from the inelastic neutron scattering intensity should be compared directly without taking a look at the supplement.

Fig.S7 and Fig.S8 and the related inelastic neutron scattering analysis in field are now included in the main text (see new Figs.5 and Fig.6c).

Similarly, scaling plots (Fig.5) provide one of the important results of the manuscript. This could also be included in the main text.

Fig.S9(a),(d)(e) are now included in the main text as Fig.7. The related discussion of quantum critical scaling has been merged into the main text as well.

3. The authors claim that that the theoretical analyses of their experimental results indicate that the Yb spins are modeled by Eq.(1). For instance, they summarize, "Both, the gapless spinon spectrum and the critical behavior around the saturation field at T>T_N unambiguously demonstrate that the intra-chain exchange is Heisenberg like to a very good approximation." On the contrary to this claim, the results just provide circumstantial evidence but not compelling. For instance, the level of exchange magnetic anisotropy could be analyzed from the shape of the wavevector-dependent neutron scattering spectra.

The shape of the neutron scattering spectra is not enough to estimate the exchange anisotropy: as shown in J. Caux et al., J. Stat. Mech. 2012, P01007 Figs. 1 and 2, for $0.6 \leq \Delta \leq 1$, the shape almost does not change.

That being said, the critical analysis clearly points to a Heisenberg universality class scaling down to 0.3K. Comparing to the exchange $J{\sim}0.21meV{\sim}2.4K$, we can still narrow down the range of anisotropy to be at least $\Delta > 0.875$ (which is a conservative estimate).

Before answering the next two comments, there is one point that we would like to clarify here because it was not emphasized enough in the original version of the manuscript. Indeed, we thank the referee for bringing this issue to our attention. *If the intra-chain Hamiltonian were an XXZ model, the uniaxial symmetry axis of such spin model would be the chain direction (which is practically orthogonal to the easy-axis z-spin direction). Given that the g-tensor is non-negligible only along a direction that is practically orthogonal to the chain direction (see Fig. 2a), the Zeeman coupling to the uniform magnetic field would break the U(1) symmetry of the XXZ model (the field is not applied along the U(1) symmetry axis of the model) leading to an Ising quantum critical point in D=1+1 dimensions at the critical field B_{sat}. Among other differences, the dynamical exponent of this QCP is z=1 instead of the z=2 value of a free fermion fixed point. It is then clear that a free fermion fixed point at B= B_{sat} is only compatible with an SU(2) invariant Heisenberg intrachain interaction. In summary, the observation of the free fermion fixed point is not just circumstantial evidence of Heisenberg interaction, but a rather compelling evidence in view of the scaling arguments that are*

now given in the main text of the manuscript. We have made this point much more explicit in the new version of the manuscript.

It could also be tested with NMR and ESR experiments.

Thanks for referee's suggestion. ESR (or EPR) is very useful for detecting the single ion anisotropy, and earlier Mössbauer effect and ESR studies have been performed by P. Bonville and his coworkers (ref.12 and ref.13 are added as the new references in the main text). The Ising single ion anisotropy of Yb (gz=6.45, gy=0.6, and gx=0.5) found by ESR in YbAlO3 and doped YAlO3 polycrystals are well consistent with our conclusions based on YbAlO3 single crystal magnetization.

On the other hand, these experiments do not work well for determining the anisotropy of exchange interaction, comparing to inelastic neutron scattering. But, an NMR or ESR experiments near the QCP maybe interesting in future for exploring the quantum critical dynamics.

It is more or less clear that the scaling behavior in the case of B>B_s should approach the free-fermion, since such a strong magnetic field as fully polarizes the spin reduces the symmetry to U(1) at most. It would be possible to extract a change in the Tomonaga-Luttinger parameter in the scaling analysis with changing magnetic field. This analysis is theoretically solid and will appreciably strengthen the case.

As we explained above, the observation of a free fermion fixed point is enough evidence of Heisenberg intra-chain interaction because the Hamiltonian symmetry would not be U(1) if the model were XXZ (the application of the field would break the U(1) symmetry). Extracting the field dependence of the Tomonaga-Luttinger parameter from the experimental data is highly non-trivial.

Also from a theoretical point of view, it would be possible to estimate the form of exchange interactions, as it has been performed for Yb2Ti2O7 (K.A. Ross et al., PRX 1, 021002 (2011); L.-J. Chang et al., Nature Comms. 3:992 (2012)) in S. Onoda, J. Phys.: Conf. Series 320, 012065 (2011).

The magnon above B_{sat} is a pure transverse mode. As explained in the manuscript, it is not possible to observe this transverse mode with INS because the neutron spin only couples to the longitudinal component (remember that the g-tensor is non-negligible only along one spin direction [$(g^{zz}/g^{xx})^2 \approx 273$],). Therefore, while it would be indeed very useful to extract the model parameter from a fit of the magnon dispersion above B_{sat}, this is not possible in YbAlO3 because transverse magnons are invisible for neutron measurements.

Any of these attempts is rather important as the authors admit that a Heisenberg interaction

between Yb magnetic moments is a surprise. It is particularly surprising when the exchange interactions have spatial anisotropy and the g-tensor is anisotropic.
The authors could choose whether they add such direct evidence to strengthen the case or reword the associated statements more carefully by commenting on the other possibilities.

As explained above, the observation of a free fermion fixed point at the saturation field is strong evidence of isotropic intra-chain Heisenberg interactions, in spite of the strong g-tensor anisotropy.

4. There are several misleading phrases and sentences:

p.5.
The phrase "the spin gap closes abruptly within a narrow temperature range of about T_N\pm0.1 K" does not make sense, since the spin gap, if it exists, means that the ground state is spin-singlet. A similar expression also appears in the Fig.3 caption. The "spin gap" should read the gap in spin exciations or the magnon/spin wave gap.

We agree with the Referee. In the new version of the manuscript we refer to this gap as the “spin excitation gap”.

p.7.
“between intrachain and intrachain interactions” is clearly odd.
We thank the Referee for pointing this out. We have corrected this sentence in the updated version of the manuscript.

Reviewers' comments:

Reviewer #1 (Remarks to the Author):

To me the most interesting outcome of the revision is the simulation of the dynamical structure factor in the disordered phase using a paramagnon picture. Of course the paramagnon picture is not uniquely
defined, but still it sheds light on the problem. Interestingly, the experimental structure factor has similaraties with the 1D spinon model and with the 3D paramagnon model. I would say that the experiment is in the middle.

I am still not convinced that the data indicate the confinement-deconfinement transition, but there is no doubts that the paper brings several important messages illuminating the problem of quantum critical
physics. I recommend the paper for publication in Nature Communications.

I would also recommend the authors to make the following clarifications
1) To articulate clearly if the temperature driven transition at T_N is "a first order like transition" or the "3D Ising model transition" with corresponding critical indexes.
2) Comment why in Fig.4 panels a,b,c there is a very significant spectral weight at \omega=0 and at any q.
3) Replace TTL to TLL and fix other misprints.

Reviewer #2 (Remarks to the Author):

The revised manuscript is well written. My questions are answered, although it is not complete because of difficulty. I recommend publication in Nat. Commun.

Reviewer #3 (Remarks to the Author):

The authors have made a lot of efforts to revise the manuscript in reply to many referee comments. Among many, however, I find some criticisms remain to be effective.

The authors added some arguments on the case of the XXZ exchange magnetic anisotropy. In particular, they commented on the maximally allowed XXZ anisotropy, which is useful if the interaction takes the XXZ form. However, actual exchange magnetic anisotropy should be more complicated, including Dzyaloshinskii-Moriya interaction and the XYZ interaction. Gapless features at higher tempeature might also be caused by some diffusive nature of the material. Then, I raised two suggestions.

ESR experiments will provide crucial information on the line width, which is significantly broadened by the exchange magnetic anisotropy in effectively spin-1/2 systems. (I'm not talking about the EPR dispersion, as the authors are, to detect the g-tensor.)

Ab initio calculations of anisotropic superexchange interactions for Yb moments, as well as symmetry arguments, would pin the form of the exchange interactions and the magnitude of the anisotropy. (See for instance, J. Phys. Conf. Series 320, 012065 (2011).)

It may be true that the possiblly strong magnetic anisotropy could be suppressed by some reasons in

this compound, the maximal XXZ anisotropy of 12 % is not that small compared to, for instance, typical Heisenberg systems such as many cuprates. It is rather hard to agree that the current scaling arguments around the saturation field provide compelling evidence for the Heisenberg interaction.

In conclusions, I do not recommend the publication of the current revised mansucript.

Response to the Referees and Changes Made in the Manuscript

Referee questions, remarks or comments are in black, our replies are in blue.
New text and changes in the manuscript are highlighted by red color.

Reply to Referee #1

To me the most interesting outcome of the revision is the simulation of the dynamical structure factor in the disordered phase using a paramagnon picture. Of course the paramagnon picture is not uniquely defined, but still it sheds light on the problem. Interestingly, the experimental structure factor has similarities with the 1D spinon model and with the 3D paramagnon model. I would say that the experiment is in the middle.

I am still not convinced that the data indicate the confinement-deconfinement transition, but there is no doubts that the paper brings several important messages illuminating the problem of quantum critical physics. I recommend the paper for publication in Nature Communications.

I would also recommend the authors to make the following clarifications
1) To articulate clearly if the temperature driven transition at T_N is "a first order like transition" or the "3D Ising model transition" with corresponding critical indexes.

Our response: Within our experimental resolution, the continuous temperature dependence of entropy S (see Fig.3c, red line) is consistent with a second order transition at T_N=0.88K. Similarly, the temperature evolution of the Bragg peak is also consistent with the second order picture (see Fig. 3c, black filled circles). However, to experimentally extract the critical exponents requires an order parameter scan using a diffraction beam line with very delicate temperature control, which will be performed in a separate future work. We emphasize that, while the order of the transition is an interesting question by itself, its answer should not affect the key message of our paper: the emergence of TLL physics from the moments with strong easy-axis anisotropy, which from a naive point of view would be classical. We have added one sentence to this effect to the last paragraph in the section "Model Hamiltonian and zero-field inelastic neutron scattering spectrum".

2) Comment why in Fig.4 panels a,b,c there is a very significant spectral weight at \omega=0 and at any q.

Our response: The original spectral weight around \omega=0 comes from the incoherent elastic scattering, as seen from the line cuts in Fig.5(d-f).
We thank Referee 1 for pointing out this issue. Our results in Fig. 4 are background-subtracted, using data measured at 2T. The residual \omega=0 spectral weight is a result of the noise in the background subtraction process, which cannot be fully eliminated. We have verified that the high-energy spectral weight is much less sensitive to this background subtraction process. Similarly, the

zero field data in Fig.3 are background-subtracted using an empty can data, to remove the direct beam scattering around Q=0. This process also lead to some small negative residual \omega=0 weight, within the window of instrumental resolution ±0.05meV (see Fig.5(d,e), and also Fig.S7 in the supporting material). In the new version of the manuscript, we have added a paragraph on the background subtraction to the method section for neutron scattering.

3) Replace TTL to TLL and fix other misprints.
We thank Referee for the careful reading! We have made the corrections in the new version of the manuscript.

Reply to Referee #2

The revised manuscript is well written. My questions are answered, although it is not complete because of difficulty. I recommend publication in Nat. Commun.

Our response: We thank the Referee for his/her careful review of the manuscript and for a positive assessment of our work.

Reply to Referee #3

The authors added some arguments on the case of the XXZ exchange magnetic anisotropy. In particular, they commented on the maximally allowed XXZ anisotropy, which is useful if the interaction takes the XXZ form. However, actual exchange magnetic anisotropy should be more complicated, including Dzyaloshinskii-Moriya interaction and the XYZ interaction. Gapless features at higher temperature might also be caused by some diffusive nature of the material. Then, I raised two suggestions.

ESR experiments will provide crucial information on the line width, which is significantly broadened by the exchange magnetic anisotropy in effectively spin-1/2 systems. (I'm not talking about the EPR dispersion, as the authors are, to detect the g-tensor.)

Ab initio calculations of anisotropic superexchange interactions for Yb moments, as well as symmetry arguments, would pin the form of the exchange interactions and the magnitude of the anisotropy. (See for instance, J. Phys. Conf. Series 320, 012065 (2011).)

It may be true that the possibly strong magnetic anisotropy could be suppressed by some reasons in this compound, the maximal XXZ anisotropy of 12 % is not that small compared to, for instance, typical Heisenberg systems such as many cuprates. It is rather hard to agree that the current scaling arguments around the saturation field provide compelling evidence for the Heisenberg interaction.

Our response: We find the comments of the Referee #3 beside the point and not helpful to proceed for the reasons listed below. Still, we have made additions to the manuscript in two places in response to points 2 and 4.

1. The magnetic field is not applied along the chain direction (rather, it is nearly perpendicular). In other words, the field is applied along a low-symmetry direction.
2. The observation of a QCP in the BEC universality class (free fermion fixed point with Z=2 at the saturation field) strongly suggests that the exchange interaction is nearly isotropic. Note that *any type of anisotropy* (symmetric or antisymmetric) will change the universality class of that QCP from BEC to Ising-like. We have added two sentences in the last paragraph before the discussion.
3. A very careful job was done to demonstrate that the saturation field QCP corresponds to a free fermion fixed point, automatically implying that any anisotropic term (XXZ or any other one) of the kind that usual appears in rare earths is small in comparison with the isotropic exchange.
4. The result mentioned in 3) is appearing in an increasing number of Yb-based materials (see references 1, 15 and 16). We have added one sentence to the section "Model Hamiltonian and zero-field inelastic neutron scattering spectrum" and one to the discussion.
5. Rau and Gingras [15] derived an isotropic super-exchange interaction for Yb-based compounds with edge-sharing octahedra. While it is not yet clear that this result applies directly to our material, it shows that Yb-based compounds can support nearly isotropic super-exchange of the type that we are finding in $YbAlO_3$.
6. One would expect a narrow ESR line for isotropic interactions (this is what Referee 3 has in mind). However, this verification is beyond the scope of our manuscript. We are already providing enough experimental evidence (INS at low fields and scaling analysis at the saturation field) in support of the nearly isotropic exchange. An ESR study will not add much to the story. The main point of our manuscript is not the isotropic super-exchange per se, but the interesting quasi-1D physics that is enabled by the combination of nearly isotropic intra-chain super-exchange and dipolar (Ising-like) inter-chain interaction. As we explain in the discussion section, this unexpected situation, which is appearing in an increasing number of Yb-based compounds, enables the study of models, which have traditionally been regarded as "toy Hamiltonians" due to the combination of interactions that do not appear together under normal circumstances. The array of TLLs coupled by density-density interactions is a clear example of a model that was originally introduced to study the possible existence of sliding TLL phases, but it is difficult to find in real materials due to the very unusual combination of intrachain XXZ exchange and pure Ising interchain interaction. This is the point that makes the paper interesting and exciting (not just another Yb-based compound with nearly isotropic exchange).

Note: The authors are responding to an editorial request.

Response to the Referees and Changes Made in the Manuscript

Referees' comment: "... we would like to see more sentences to explicitly acknowledge that further experiments such as ESR would help clarify the isotropic interactions and also to mention that the result in Ref [15] is not directly applicable to your material."

We added the following paragraph to the Discussion section:

Rau and Gingras \cite{Rau18} derived an isotropic super-exchange interaction for Yb-based compounds with edge-sharing octahedra. While it is not yet clear that this result applies directly to our material, it shows that Yb-based compounds can support nearly isotropic super-exchange of the type that we are finding in YbAlO$_3$. Also, note that further experiments such as an electron spin resonance would help quantify the small anisotropies in this system.
